# Targeting ferroptosis as a therapeutic strategy for hepatotoxicity

**DOI:** 10.1016/j.toxrep.2025.102137

**Published:** 2025-10-10

**Authors:** Negar Hemmati, Mahdieh Anoush, Bahman Abedi Kiasari, Alireza Torkamani

**Affiliations:** a Department of Microbiology and Immunology, Faculty of Veterinary Medicine, University of Tehran, Tehran, Iran; b Zanjan Applied Pharmacology Research Center, Health and Metabolic Diseases Research Institute, Zanjan University of Medical Sciences, Zanjan, Iran; c Department of Pharmacology and Toxicology, School of Pharmacy, Zanjan University of Medical Sciences, Zanjan, Iran; d School of Metallurgy and Materials Engineering, Iran University of Science and Technology (IUST), Tehran, Iran

**Keywords:** Hepatotoxicity, Ferroptosis, Iron Homeostasis, Therapeutic Strategies, Oxidative Stress

## Abstract

Hepatotoxicity is a global health problem with a scarcity of treatment modalities that can reverse extensive liver injury. The liver’s central role in detoxification, iron regulation, and redox balance makes it highly vulnerable to toxic, metabolic, and inflammatory insults. Ferroptosis, an iron-dependent form of regulated cell death, has emerged as a key mechanism linking oxidative stress, disrupted iron homeostasis, and hepatocellular injury Ferroptosis differs from apoptosis or necroptosis since it entails metabolic frailties of the glutathione–GPX4 system, the FSP1–CoQ10 system, and the BH4/DHFR system with peroxidation failure-prone lipid remodeling processes. Notably, growing evidence implicate ferroptosis in a number of hepatic disorders, including drug-induced liver injury, alcoholic and non-alcoholic liver disease, ischemia–reperfusion injury, and non-alcoholic steatohepatitis. Beyond acute death of hepatocytes, chronic ferroptotic signaling also enhances fibrogenesis and accelerates cirrhosis and hepatocellular carcinoma, and viral hepatitis exploits ferroptotic mechanisms via iron imbalances and oxidative stress. Acting both sides of this coin, pathogenic catalyst of parenchymal disease and therapeutic target of malignancy, puts ferroptosis both as a liability and a target. New therapeutic modalities, including natural products, synthetic small molecules, mesenchymal stem cell derivatives, and nanoparticle systems, modulate ferroptotic signaling to enhance antioxidant defenses, restore iron balance, or selectively induce tumor cell death. Collectively, these strategies underscore the translational promise of ferroptosis-based interventions. This review integrates mechanistic insights with emerging therapies, positioning ferroptosis as a context-dependent driver of hepatotoxicity and a compelling target for precision liver medicine.

## Highlights

• Ferroptosis is a regulated, iron-dependent form of cell death that plays a central role in hepatotoxicity.
• Dysregulated iron homeostasis and impaired antioxidant defenses sensitize hepatocytes to ferroptotic injury.
• Ferroptosis is implicated in a wide range of liver disorders.
• Emerging therapeutic approaches aim to modulate ferroptosis for liver protection.
• Targeting ferroptosis offers hepatoprotective and precision therapeutic potential.

## Graphical Abstract

**Figure:**
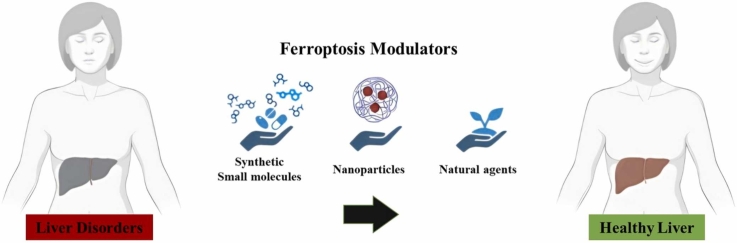

## 1 Background & overview

Hepatotoxicity is a critical contributor to global liver-related morbidity and mortality [1], [2], [3]. The liver’s central role in detoxification, nutrient processing, and systemic iron regulation makes it particularly vulnerable to diverse insults, including xenobiotics, alcohol, ischemia-reperfusion stress, and metabolic dysfunction [4], [5], [6]. Clinical entities such as drug-induced liver injury (DILI), alcohol-associated liver disease (ALD), and ischemia-reperfusion injury (IRI) represent leading causes of acute and chronic hepatic damage, frequently culminating in cirrhosis, organ failure, or transplantation. In parallel, the rising prevalence of non-alcoholic steatohepatitis (NASH) underscores the growing burden of metabolic hepatotoxicity. Collectively, these disorders highlight the urgent need to clarify mechanisms of hepatic injury in order to guide the development of targeted interventions [7], [8], [9], [10]. A central determinant of liver pathology is the nature of cell death activated in hepatocytes, beyond classical cell death pathways, such as apoptosis, autophagy, and pyroptosis, a newer form known as ferroptosis has gained attention. Ferroptosis is an iron-dependent process characterized by the accumulation of lipid peroxides [11]. Several metabolic pathways are implicated in regulating ferroptosis, including the FSP1/coenzyme Q axis, the GCH1/BH4/DHFR axis, and the cysteine /GSH /GPX4 axis. Emerging evidence suggests ferroptosis plays a critical role in the progression of various liver disorders, including DILI, ALD, IRI, and metabolic fatty liver disease. Importantly, ferroptosis also contributes to the pathogenesis of hepatocellular carcinoma (HCC), where its induction may restrict tumor growth but resistance mechanisms often emerge to sustain malignancy [11], [12], [13], [14]. From these findings, therapeutic targeting of ferroptosis seems to be a viable option [15]. Several interventions are currently being explored, including natural products [16], synthetic molecules, MSCs and MSC-derived exosomes [17], [18], Nano Particles [19], retinoic acid [20], melatonin [21], and maresin [22]. These therapies exhibit potential in reducing liver damage through the regulation of ferroptosis signaling pathways. This review explores the current understanding of ferroptosis in the context of hepatotoxicity with diverse causes and etiologies. We also highlight recent advances in the therapeutic landscape, focusing on iron metabolism, mechanisms of hepatic cell death, and innovative treatments, including natural products, NPs, and MSC-based therapies.

## 2 Ferroptosis and hepatic metabolism

**Fig. 1:**
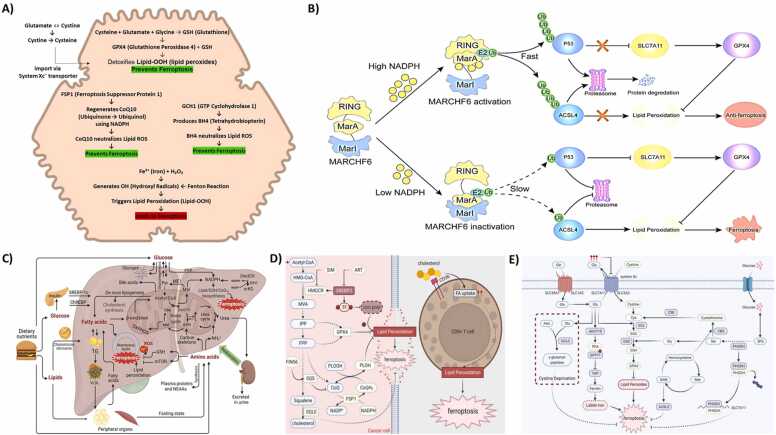
Metabolic control points that tune ferroptosis in the liver.**(A)** Key intracellular pathways regulating ferroptosis in hepatocytes. Anti-ferroptotic defenses include system x_c⁻–GSH–GPX4, FSP1–CoQ10–NAD(P)H, and GCH1–BH4–DHFR axes that detoxify phospholipid hydroperoxides. Pro-ferroptotic drivers comprise labile Fe²⁺ (Fenton chemistry), ACSL4/LPCAT3-mediated PUFA-phospholipid synthesis, and LOX/POR/NOX-catalyzed lipid peroxidation; membrane repair by ESCRT-III provides transient protection but can be overwhelmed. **(B)** NADPH sensing by MARCHF6. Schematic of the ER E3 ligase MARCHF6 acting as a cytosolic NADPH sensor: at high NADPH, MarA–RING engagement accelerates ubiquitin-dependent degradation of p53 and ACSL4, thereby favoring SLC7A11/GPX4 expression and suppressing lipid peroxidation; at low NADPH, MarI–MarA interaction dampens ligase activity, stabilizes p53/ACSL4, and licenses ferroptosis [35] (CC BY 4.0). **(C)** Hepatic metabolic integration. The liver coordinates glucose, lipid, and amino-acid metabolism: post-prandial glucose is stored as glycogen and routed through glycolysis and the PPP to generate NADPH, with ME1 and IDH1 providing additional NADPH. Excess carbohydrate drives de novo lipogenesis; during fasting, glycogenolysis and gluconeogenesis maintain glycemia. Lipid handling includes clearance of chylomicron remnants and assembly of VLDL. Amino-acid turnover (deamination/transamination) feeds ureagenesis. On the ferroptosis axis, SLC7A11-mediated cystine uptake supports GSH, and mTORC1 signaling tunes GPX4. Dysregulated macronutrient flux contributes to insulin resistance, NAFLD/NASH, and cholestasis [25] (CC BY 4.0). **(D)** Mevalonate/sterol control of ferroptosis. The mevalonate (MVA) pathway (via HMGCR, SQS, SQLE) produces IPP/FPP for selenocysteine-tRNA isopentenylation (efficient GPX4 biosynthesis) and CoQ10 generation; SREBP2 coordinates this program. Pharmacological perturbations (e.g., statins, FIN56) remodel CoQ10 pools and ferroptosis sensitivity. In the tumor microenvironment, cholesterol can upregulate CD36 on CD8⁺ T cells, heightening FA uptake and lipid peroxidation, thereby driving T-cell ferroptosis [36] (CC BY 4.0). **(E)** Amino-acid checkpoints. Glutamate, cysteine, and glycine constitute GSH. Glutamine supplies glutamate; cystine enters via system x_c⁻ (SLC7A11/SLC3A2) and is reduced to cysteine; high extracellular glutamate inhibits x_c⁻. Serine–glycine/one-carbon metabolism (e.g., PHGDH) produces cysteine and glycine; transsulfuration (CBS/CSE) provides a backup cysteine source. These nodes regulate SLC7A11, the labile iron pool, and GPX4, thereby shaping ferroptosis susceptibility [36] (CC BY 4.0).

The liver is the central organ of metabolic regulation and serves a critical function in glucose, lipid, and amino acid metabolism [23] (Fig. 1C). Disbalance among those macronutrients may cause oxidative stress, liver damage, and, in severe conditions, life-threatening consequences [24]. Recent studies have placed growing emphasis on the function of ferroptosis, a regulated, iron-catalyzed process of cell death, in numerous metabolic pathways in liver. One of the key associations refers to the impact of ferroptosis on the levels of nicotinamide adenine dinucleotide phosphate (NADPH), Beyond serving as a biomarker of ferroptosis, hepatic NADPH metabolism is directly shaped by three central enzymatic pathways: the pentose phosphate pathway (PPP), malic enzyme 1 (ME1), and cytosolic isocitrate dehydrogenase 1 (IDH1) [25], [26], [27]. These pathways maintain the reducing power required for glutathione (GSH) and coenzyme Q10 (CoQ10) synthesis, both potent ferroptosis inhibitors. In addition to metabolic flux, transcriptional regulation contributes to NADPH homeostasis. NRF2, dissociating from KEAP1 under oxidative stress, induces antioxidant response element (ARE)-driven genes that regenerate NADPH and modulate iron-related proteins (FTH1, FTL1, SLC40A1, HMOX1), thereby suppressing ferroptosis. More recently, studies identified MARCHF6 as an ER-resident ubiquitin ligase that functions as an intracellular NADPH sensor [28], [29], [30], [31], [32]. High NADPH levels enhance MARCHF6 ligase activity, promoting ubiquitin-dependent degradation of pro-ferroptotic factors such as p53 and ACSL4, while upregulating GPX4 and SLC7A11 [33]. Conversely, NADPH depletion abrogates this mechanism, driving lipid peroxidation and ferroptosis [34]. Importantly, in vivo MARCHF6 deletion led to severe hepatic injury and perinatal lethality in mice, which were partly rescued by lipophilic antioxidants, highlighting the therapeutic relevance of the NADPH–MARCHF6 axis in liver physiology and ferroptosis regulation [27], [35] (Fig. 1B).

Lipid metabolism is the most relevant to ferroptosis since its hallmark is the generation of lipid peroxides [36], [37], [38]. Initiation of lipid peroxidation can arise from multiple oxidant sources, including NADPH oxidases (NOX), cytochrome P450 reductase (POR), and NADH–cytochrome b5 reductase (CYB5R1), all highly relevant in hepatocytes given their dense CYP system. The role of lipoxygenases (LOXs) remains context-dependent but may catalyze direct PUFA oxidation in certain settings [36], [39]. Once lipid hydroperoxides form, reaction with ferrous iron (Fe²⁺) generates peroxyl radicals that abstract bis-allylic hydrogens from neighboring PUFA chains, driving propagation [40]. Arachidonoyl- and adrenoyl-containing phosphatidylethanolamines are prominent pro-ferroptotic substrates, whereas incorporation of monounsaturated fatty acids (MUFAs) into membranes antagonizes peroxidation [41]. Spatially, early peroxide accumulation occurs at the endoplasmic reticulum, progressing to the plasma membrane where threshold breach results in catastrophic permeabilization, despite transient repair attempts by the ESCRT-III complex (Fig. 1**A**) [42]. Notably, EFAs when aberrantly metabolized, are directly responsible for inflicting oxidative damage on liver tissues [43], [44], [45], [46]. This is best observed in conditions like non-alcoholic fatty liver disease (NAFLD), a condition of pathological storage of lipids in the liver. If unchecked, NAFLD will progress into NASH, an aggravated, fibrotic form of liver disease characterized by inflammation and cell death [47]. Hepatic ferroptosis sensitivity is critically shaped by lipid handling pathways [34]. The ACSL4–LPCAT3 axis channels PUFAs into phospholipids to promote ferroptosis, while ACSL3-mediated MUFA incorporation confers resistance [48], [49]. SCD1, under SREBP1 control, drives MUFA synthesis (oleate, palmitoleate), dampening ferroptosis; this program is reinforced by mTORC1 activation and suppressed by AMPK [50]. Ether lipid metabolism also contributes, with AGPS and TMEM164 generating PUFA-ether phospholipids that, in certain hepatocellular contexts, are indispensable for ferroptosis [51], [52], [53]. Conversely, catabolic enzymes such as DECR1 limit PUFA accumulation via mitochondrial β-oxidation, and their loss amplifies ferroptotic vulnerability [34], [54]. Lipid uptake and storage further modify susceptibility: CD36/FATP transporters deliver exogenous fatty acids, while sequestration into lipid droplets via TPD52 provides protection, opposed by lipophagy-mediated PUFA release such as PGRMC1 [55], [56]. These mechanisms intersect with physiological hepatic lipid fluxes, VLDL assembly, phospholipid remodeling, and ER oxidative metabolism, explaining why PUFA-enriched states such as NAFLD/NASH expand the ferroptotic window. From a therapeutic perspective, ferroptosis can be suppressed by radical-trapping antioxidants (ferrostatin-1, liproxstatin-1 and derivatives) or, conversely, induced by PUFA supplementation, GPX4/FSP1 inhibition, or SCD blockade, highlighting clinical opportunities to either mitigate oxidative liver injury or trigger ferroptotic death in hepatocellular carcinoma (Fig. 1B). Beyond fatty-acid composition, the mevalonate pathway modulates ferroptosis by enabling GPX4 translation and maintaining CoQ10 pools. Cholesterol metabolism provides a parallel, and intimately connected, layer of control through the mevalonate (MVA) pathway [57]. Beginning with acetyl-CoA and the rate-limiting enzyme HMG-CoA reductase (HMGCR), the MVA cascade generates isopentenyl pyrophosphate (IPP) [37]. IPP serves two ferroptosis-critical purposes: (i) it is required for isopentenylation of selenocysteine tRNA, a prerequisite for efficient GPX4 biosynthesis; and (ii) it feeds ubiquinone (CoQ) production, whose reduced form (ubiquinol) is regenerated by FSP1 at membranes to quench peroxyl radicals. Accordingly, pharmacologic or transcriptional brakes on MVA flux lower GPX4 and/or CoQ availability and sensitize cells to ferroptosis, whereas robust MVA output fortifies membranes against lipid autoxidation [58], [59] (Fig. 1D).

Amino acids also play a central role in ferroptosis regulation, especially in their function to modulate oxidative stress responses [14], [60], [61]. Cystine import via system x_c⁻ (SLC7A11/SLC3A2) and backup transsulfuration (CBS/CSE) supply cysteine for GSH; blocking either axis collapses peroxide detoxification and licenses PUFA-PL peroxidation. The serine–glycine–one-carbon network feeds GSH synthesis and NADPH generation, stabilizing GPX4; limiting serine/glycine or one-carbon flux sensitizes cells. Glutamine is context-dependent: it fuels x_c⁻ exchange and NADPH production but, under cystine scarcity, can drive oxidative metabolism that accelerates lipid peroxidation. Arginine sensing (mTORC1/GCN2–ATF4) tunes this landscape co-depletion of arginine blunts cystine-starvation-induced ferroptosis by downshifting anabolic/redox demand [62], [63], [64]. Interestingly, one of Conlon et al.'s studies had demonstrated that merely the absence of cystine alone was not sufficient to induce ferroptosis if there wasn't a deficiency in other amino acids, like arginine [65], [66], [67]. This is an indication of the presence of more complex interaction between amino acids and how it controls ferroptosis sensitivity (Fig. 1**E**).

## 3 Iron homeostasis

### 3.1 Cellular iron homeostasis

Iron balance is carefully regulated at the cellular level by the interaction of Iron Regulatory Proteins (IRPs) and Iron-Responsive Elements (IREs) found in the untranslated regions of mRNA transcripts related to iron metabolism [68], [69], [70]. This process of post-transcriptional regulation is crucial for maintaining cellular iron homeostasis, allowing cells to adjust their iron uptake, storage, and export based on the available iron levels. In conditions where iron is deficient, IRP1 and IRP2 enhance iron acquisition by stabilizing the mRNA of transferrin receptor 1 (TFR1) while simultaneously blocking the translation of ferritin and ferroportin, which diminishes iron storage and export [70], [71]. IRP1 becomes activated in iron-replete cells by incorporating iron-sulfur (Fe/S) clusters, while IRP2 degrades via iron-dependent proteasomes. This dual regulation significantly controls iron metabolism and links iron availability to essential metabolic pathways, such as the tricarboxylic acid (TCA) cycle [72]. IRP1 is highly active in hypoxic environments, such as the kidney and duodenum, while IRP2 operates in tissues with stable oxygen levels. Animal studies, primarily using mice with specific or complete deletions of the IRP proteins, have been vital in demonstrating the critical functions of these proteins [73], [74]. Mice lacking both IRPs do not survive, highlighting their essential role in maintaining iron balance. The loss of IRP2 alone in both humans and mice can lead to adult-onset neurodegenerative disorders, erythropoietic protoporphyria, and mild anemia, likely due to insufficient functional iron. Interestingly, while adult IRP1-deficient mice appear normal in standard conditions, deleting both IRPs in intestinal epithelial cells results in iron malabsorption and localized iron retention caused by ferritin accumulation [69], [75], [76]. Certain cell types, such as erythroblasts and enterocytes, produce a version of ferroportin that lacks a 5′ IRE. This allows for continuous iron export even in deficiency, although hepcidin still regulates it. Another important factor is nuclear receptor co-activator 4 (NCOA4), which controls ferritinophagy, the process by which ferritin is broken down to release stored iron during periods of iron deprivation. NCOA4 is regulated post-translationally and is degraded in iron-rich environments by the E3 ubiquitin ligase HERC2 [77], [78]. NCOA4 facilitates ferritin degradation in iron-depleted cells, linking iron availability to DNA replication, cellular senescence, and replication stress. A deficiency in NCOA4 can lead to an accumulation of iron in organs such as the liver and spleen, impaired iron recycling, and increased susceptibility to anemia. Elevated NCOA4 levels in erythroid cells emphasize its crucial role in hemoglobin synthesis, as shown in studies involving mouse models, zebrafish, and in vitro experiments [77], [79], [80]. However, the primary systemic importance of NCOA4 lies in maintaining iron balance through ferritinophagy in macrophages, which recycle iron and support overall iron homeostasis [81].

### 3.2 Systemic iron homeostasis

**Fig. 2:**
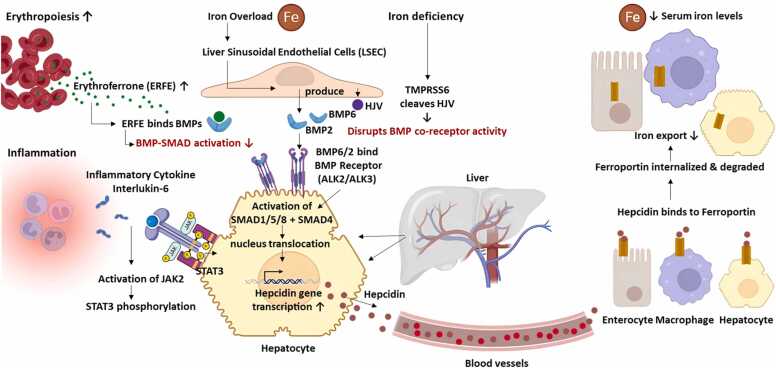
Integrated regulation of hepcidin production and systemic iron homeostasis under inflammatory, iron-deficiency, and iron-overload states**.** Iron overload stimulates liver sinusoidal endothelial cells to release BMP6/BMP2, which via HJV as a co-receptor, activate BMP receptors (ALK2/ALK3) and the SMAD1/5/8-SMAD4 pathway, driving transcription of the HAMP gene in hepatocytes. In parallel, inflammation induces IL-6/JAK2/STAT3 signaling, synergizing with BMP–SMAD to upregulate hepcidin. Conversely, iron deficiency activates TMPRSS6 (matriptase-2), which cleaves HJV and suppresses BMP co-receptor activity, thereby dampening hepcidin synthesis. Secreted hepcidin binds to ferroportin on enterocytes, macrophages, and hepatocytes, triggering ferroportin internalization and lysosomal degradation, ultimately lowering plasma iron availability. This axis links iron sensing, inflammatory cues, and hepatic transcriptional control to systemic iron distribution.

At the systemic level, the hormone hepcidin is crucial in regulating iron metabolism, positioning the liver as a key organ. Hepcidin strictly controls the amount of iron in the plasma by binding to ferroportin, the only known cellular iron exporter [82], [83]. This binding results in the internalization and degradation of ferroportin in enterocytes, hepatocytes, and macrophages. Such tight regulation limits the export of iron into the bloodstream, highlighting the essential role of hepcidin in maintaining systemic iron homeostasis [83], [84]. Hepcidin production in hepatocytes increases by circulating and tissue iron levels through signaling from liver sinusoidal endothelial cells. These endothelial cells produce BMP6 and BMP2, activating the BMP-SMAD pathway in hepatocytes, leading to increased hepcidin transcription [82], [85]. BMP6 expression is responsive to iron levels and may be regulated by the NRF2-mediated antioxidant pathway, while BMP2 is consistently expressed and less sensitive to iron status. BMP6 and BMP2 play crucial roles in regulating hepcidin, with BMP6 being more reactive to changes in iron levels [85], [86], [87]. Additionally, Inflammatory stimuli, such as interleukin-6 (IL-6), activate the JAK2-STAT3 signaling pathway, further enhancing hepcidin expression [88]. This enhanced expression is particularly relevant in the anemia of inflammation, where elevated hepcidin levels restrict iron availability despite sufficient or even elevated iron stores [89], [90], [91]. IL-6 significantly contributes to this condition by activating the JAK2-STAT3 signaling cascade, which increases hepcidin expression and leads to iron sequestration and anemia [92], [93]. In contrast, hepcidin expression is suppressed by conditions such as anemia, hypoxia, increased erythropoiesis, higher testosterone levels, and other unidentified factors [94], [95], [96]. Matriptase-2, encoded by TMPRSS6, is an important inhibitor that lowers hepcidin levels by cleaving hemojuvelin, a BMP co-receptor [97], [98]. This cleavage disrupts BMP-SMAD signaling and plays a key role in the regulation of hepcidin [99], [100]. Additionally, regulators such as FKBP12 inhibit the activation of the BMP signaling pathway by binding to the BMP receptor ALK2 [101], [102]. Erythroferrone (ERFE), produced by erythroid precursors in response to erythropoietin, also suppresses hepcidin to promote iron availability for hemoglobin synthesis [103], [104]. Other potential modulators, including soluble hemojuvelin and platelet-derived growth factor-BB, have been observed in vitro, but their roles in vivo are still uncertain [105], [106]. In inflammatory conditions, hepcidin derived from macrophages may enhance systemic iron sequestration [107] (Fig 2).

## 4 Dysregulated iron metabolism triggers ferroptosis

Ferroptosis is a distinct form of regulated cell death characterized primarily by iron-catalyzed oxidative damage [108]. The Fenton reaction is Central to this process, where iron plays a crucial role in producing reactive oxygen species (ROS), leading to lipid peroxidation [109]. The oxidation of both enzymatic and non-enzymatic polyunsaturated fatty acids within cellular membranes is a key driver of the fatal oxidative damage associated with ferroptosis [110], [111]. A critical ferroptosis regulator is CISD1 (CDGSH iron-sulfur domain-containing protein 1), also known as MitoNEET [112], [113]. This protein inhibits ferroptosis by preventing mitochondrial iron overload. By modulating the iron content in mitochondria, CISD1 is a protective mechanism against the initiation of ferroptotic cell death [114].

Recent studies have shed light on iron's role in liver disease. The SLC39A14 transporter, which carries non-transferrin-bound iron (NTBI), has been shown to induce iron-dependent ferroptosis in mouse models lacking the transferrin receptor (Trf⁻/⁻) [115], [116]. This mechanism significantly contributes to liver fibrosis and cirrhosis, highlighting a previously underappreciated link between imbalanced iron uptake and chronic liver damage [25], [117]. Iron overload from excessive acquisition or impaired storage sensitizes cells to ferroptosis [118]. This has been demonstrated using the ferroptosis-inducing agent elastin in model systems, where iron supplements and chelators influence cell vulnerability [119]. Ferritinophagy, an NCOA4-dependent selective autophagy process, breaks down ferritin and redistributes stored iron into the cytosol [77]. This release of free iron promotes lipid peroxidation, further exacerbating ferroptotic damage [120]. On the other hand, cellular defence mechanisms, such as the iron chaperone PCBP1 (Poly(rC)-binding protein 1), are essential for maintaining iron homeostasis and preventing toxic accumulation [121]. PCBP1 safely delivers iron to enzymes and storage proteins, thereby buffering against oxidative stress and ferroptosis prompted by excess iron [122], [123]. These findings illustrate how ferroptosis is closely linked to iron metabolism, creating a feedback loop that worsens liver injury when iron balance is disrupted.

## 5 Mitochondrial GPX4 and DHODH suppress ferroptosis

**Fig. 3:**
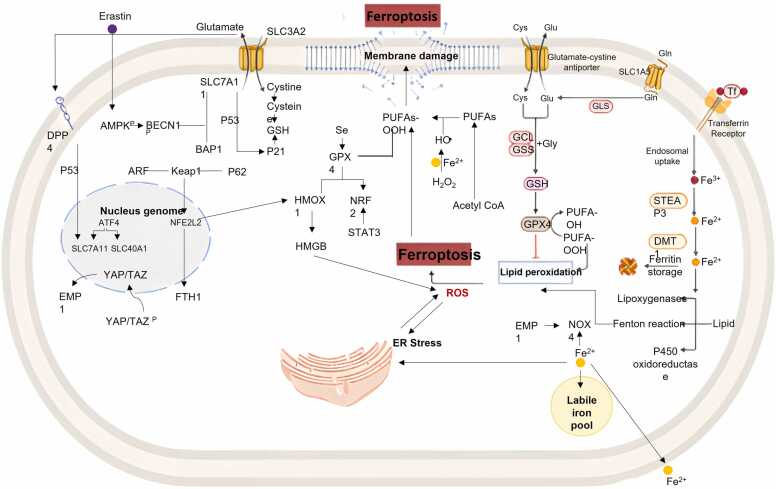
Signaling pathways and regulation of ferroptosis. Ferroptosis is a regulated, iron-dependent cell death driven by lipid peroxidation and loss of membrane integrity. Multiple signaling networks converge to control its initiation and execution. The system Xc⁻ transporter (SLC7A11/SLC3A2) maintains intracellular cysteine for GSH biosynthesis, sustaining GPX4 activity and preventing peroxidation of PUFAs. Stress sensors such as p53, BECN1, and AMPK modulate SLC7A11 expression and GSH metabolism. Iron homeostasis is regulated by transferrin-mediated uptake of Fe³ ⁺ via TFR1, reduction by STEAP3, cytosolic release through DMT1, storage in ferritin (FTH1/FTL), and efflux via SLC40A1; ferritinophagy liberates Fe²⁺ from ferritin, enlarging the labile iron pool that fuels Fenton chemistry and ROS formation. Enzymes including lipoxygenases, ACSL4, LPCAT3, NOX4, and cytochrome P450 oxidoreductase catalyse peroxidation of membrane PUFAs, while ER stress and PERK signalling influence GPX4 stability and lipid redox balance. Crosstalk among antioxidant defences, iron metabolism, and lipid-oxidising enzymes determines cellular susceptibility to ferroptosis.

Mitochondria are essential centers for cellular metabolism and energy production, primarily through the electron transport chain and oxidative phosphorylation processes [124], [125]. They play a critical role in generating ATP, but this process also leads to the unavoidable production of ROS [125], [126]. Mitochondria are one of the primary sources of ROS in cells, which can cause oxidative damage under certain pathological conditions or stresses [127]. One of the most harmful consequences of excessive ROS production is lipid peroxidation, particularly when ROS interact with polyunsaturated fatty acids (PUFAs) in cellular membranes [128], [129]. This lipid peroxidation is a key hallmark of ferroptosis, a regulated form of cell death [130]. Recent studies in metabolomics, pharmacology, and molecular biology underscore that mitochondrial metabolic activity is a significant source of ROS and essential for creating the oxidative environment that triggers ferroptosis [131], [132], [133]. Cells rely on antioxidant defence mechanisms to repair or neutralize peroxidized lipids and combat potentially lethal oxidative stress. Three crucial protective systems have been identified: GPX4, FSP1, and DHFR [25], [134], [135]. These systems act at different cellular locations to mitigate the damage caused by lipid peroxides and maintain redox homeostasis [136]. Additionally, to these well-characterized defenses, mitochondrial GPX4 and DHODH have recently emerged as important mitochondrial enzymes that help suppress ferroptosis, according to groundbreaking research by Mao et al., GPX4 and DHODH function within the mitochondria to detoxify lipid peroxides, preventing mitochondrial damage and subsequent cell death [137], [138]. DHODH is particularly noteworthy; it is a flavin-dependent, iron-containing enzyme that links the mitochondrial respiratory chain with de novo pyrimidine biosynthesis. Its dual role in metabolism and redox control makes it a potential therapeutic target especially in tumors deficient in GPX4, where inhibiting DHODH could shift the balance toward ferroptosis as a selective anticancer strategy [138], [139], [140]. These findings enhance our understanding of ferroptosis regulation and emphasize the mitochondria as a crucial battleground in the ongoing conflict between cell survival and death under oxidative stress conditions (Fig. 3).

## 6 Ferroptosis in hepatopathy

Cumulating evidence links ferroptosis with the pathogenesis of major liver diseases, both infectious and non-infectious liver diseases [25], [141], [142]. Pharmacological interruption of the peroxidation cascade (radical-trapping antioxidants such as ferrostatin-1/luproxstatin-1), enhancing endogenous antioxidant pathways such as α-tocopherol, and chelating iron each reduces tissue damage in preclinical models [25], [143]. These suggest inhibition of ferroptosis as a means of hepatoprotection in situations in which transient bursts of oxidation convert sublethal stress into membrane-catastrophic lipid peroxidation [117]. Second, in chronic disease, persistent iron loading, inflammatory signaling, and lipidomic remodeling conspire by opening the ferroptotic window. Two, seemingly mutually exclusive, therapeutic deductions follow. Firstly, inhibition of ferroptosis in hepatocytes may limit parenchymal attrition and blunt the transition from injury through fibrosis and cirrhosis. Secondly, cell-type specificity matters: cell-type selective induction of ferroptosis in hepatic stellate cells (HSCs) has been demonstrated with potential for the treatment of fibrosis, with small molecules such as artesunate and magnesium isoglycyrrhizinate described as driving active HSCs into ferroptotic cell death [144], [145]. The challenge, and opportunity, lies in the delivery of modality and dose by which this vulnerability may be used in HSCs without injuring hepatocytes [146].

### 6.1 Non-alcoholic steatohepatitis

Non-alcoholic steatohepatitis, the progressive form of non-alcoholic fatty liver disease (NAFLD), is defined by intracellular lipid accumulation, inflammation, hepatocellular ballooning, and fibrosis [147]. Growing evidence implicates ferroptosis, a regulated form of iron-dependent cell death that is mediated through lipid peroxidation, a primary mediator of NASH pathogenesis and progression [25], [148], [149]. Metabolic dysregulation, the hallmark of NASH, causes ferroptotic susceptibility through oxidative stress, glutathione depletion, and over-oxidation of PUFA [150], [151]. Researches have demonstrated that inhibition of the cystine/glutamate antiporter system Xc⁻ (by SLC7A11 inhibition), NADPH depletion, and reduced GPX4 activity reduce the cellular antioxidant defense, promoting uncontrolled lipid peroxidation in hepatocytes [152]. Mitochondrial dysfunction further increases oxidative stress, reactive oxygen species increasing ferroptotic cascades [153]. Experimental NASH models have increased hepatic iron overload and induction of ACSL4 and ALOX12/15, lipid peroxidation promoting enzymes, showing a mechanistic link between ferroptosis and severity of disease [154], [155]. Furthermore, ferroptosis also promotes NET formation in NASH, sustaining chronic inflammation and fibrogenesis [156]. Therapeutically, ferroptosis has been of interest to suppress NASH progression [149], [157]. Melatonin and ginsenoside Rd exhibit hepatoprotection through upregulation of GPX4 expression and NRF2/HO-1 axis regulation [158], [159]. Ferroptosis inhibitors ferrostatin-1, and natural compounds baicalein and epigallocatechin gallate, decreased lipid peroxidation and liver injury in models of NASH [160], [161], [162]. These findings position ferroptosis not only as a pathogenic driver but also as a promising therapeutic target in NASH. Future clinical trials are needed to validate ferroptosis-modulating strategies in human NASH and assess their long-term safety and efficacy. Recently, the terminology of NAFLD has been redefined as metabolic dysfunction–associated steatotic liver disease (MASLD), highlighting systemic metabolic derangements rather than alcohol abstinence as the primary driver of pathology [163], [164]. Mechanistically, MASLD provides increased ferroptotic susceptibility through dysmetabolic iron overload syndrome, ACSL4–LPCAT3–mediated PUFA introduction, deficient SCD1/NRF2–GPX4 antioxidant defense, and sustained inflammatory signalization. Together, these factors distinguish MASLD from simple steatosis and justify its evolution to steatohepatitis, fibrosis, and cirrhosis. Preclinical models demonstrate ferroptosis inhibitors, chelators of iron, and natural antioxidants correct redox equilibrium in MASLD, highlighting ferroptosis both as a mechanistic pivot point and therapeutics target across the metabolic continuum of fatty liver disease [151], [165], [166], [167].

### 6.2 Alcoholic liver disease

Ferroptosis has recently gained attention regarding its role in the pathogenesis of ALD despite lipid dysregulation and hepatic iron overload being established features [117], [168]. During the recovery phase of ethanol-induced liver injury, ferroptosis is typically negatively regulated [169]. Experimental studies using cell cultures and animal models have demonstrated that ethanol exposure produces a significant accumulation of ROS and lipid peroxidation, hallmarks of ferroptosis [170], [171], [172], [173], [174]. The application of ferroptosis inhibitors mitigates these effects, suggesting a causal relationship. Dimethyl fumarate has been identified as a protective agent against ethanol-induced oxidative stress [175], [176]. It achieves this by inhibiting ferroptosis and activating the Nrf2 antioxidant pathway [177], [178]. Genetic studies have further indicated that ferroptosis mediates alcohol-induced liver injury through specific gene pathways, including one involving frataxin [179], [180]. Moreover, the interaction between the liver and other organs, particularly the gut and adipose tissue, has been implicated in the progression of ALD through ferroptotic mechanisms [168], [181]. For instance, intestinal sirtuin 1 (SIRT1) impairment have been shown to reduce ferroptosis and correct the alcohol-induced iron imbalance, thereby protecting the liver [182], [183]. Another study found that overexpression of lipid-1 in adipose tissue exacerbated alcoholic steatohepatitis and hepatobiliary injury by promoting iron accumulation and lipid peroxidation, independent of GPX4 activity [184], [185]. These findings highlight the necessity for further research into ferroptosis as a potential therapeutic target in ALD.

### 6.3 Drug-induced liver injury

DILI liver injury can range from abnormal liver enzyme levels to acute liver failure and may be classified as idiosyncratic or intrinsic [186]. The intrinsic type, often resulting from acetaminophen (APAP) overdose, is more common and better studied in both human and animal models. APAP is metabolized into N-acetyl-p-benzoquinone imine (NAPQI), a reactive compound that depletes GSH and inhibits GPX4, promoting ferroptosis [186], [187], [188]. This hypothesis is supported by studies showing that the ferroptosis inhibitor ferrostatin-1 improves cell survival and liver histology in hepatocytes and mice treated with APAP [189], [190]. Although ferrostatin-1 does not restore GSH levels or inhibit NAPQI formation, it significantly reduces lipid peroxidation, suggesting that ferroptosis plays a role in APAP toxicity [191], [192]. Mitochondrial dysfunction and nitrosative stress (peroxynitrite formation) are central events in APAP-induced necrosis. Ultrastructural features such as condensed mitochondria with reduced cristae overlap with ferroptotic morphology. In parallel, release of labile Fe²⁺ from mitochondria and heme stores amplifies Fenton-driven ROS production, providing a mechanistic link between necrosis and ferroptosis. Notably, N-acetylcysteine (NAC), the clinical antidote, restores GSH but does not directly suppress iron-dependent lipid peroxidation, which may explain why ferroptosis inhibitors confer additional hepatoprotection in preclinical models [193], [194], [195].

Inhibiting ACSL4, a key enzyme in PUFA metabolism, alleviated APAP-induced hepatotoxicity by reducing lipid peroxidation and ROS levels [48], [196]. However, diets rich in PUFAs may further sensitize hepatocytes to oxidative injury, with auto-oxidation rather than enzymatic lipid peroxidation potentially driving this damage [197], [198]. Nonetheless, the role of ferroptosis in DILI remains contentious [199]. Some studies, such as Knight et al., argue that lipid peroxidation does not contribute to APAP toxicity, and antioxidant treatments like α-tocopherol do not offer protection [200]. Others suggest that APAP toxicity exhibits characteristics of prescribed necrosis distinct from ferroptosis. Consequently, ferroptosis has not yet been firmly established as a therapeutic target in DILI [192], [201], [202]. Nevertheless, accumulating evidence suggests that ferroptosis may act as a complementary pathway alongside necrosis in APAP hepatotoxicity, warranting further mechanistic and translational studies. Several natural compounds, including abietic acid, clausenamides, astaxanthin, kaempferol, and ginsenosides, have been shown to mitigate APAP-induced ferroptosis primarily by activating the Nrf2-associated signaling pathway [203], [204], [205], [206], [207]. Nevertheless, some studies contend that iron-dependent lipid peroxidation is only a secondary driver of liver injury under normal dietary conditions, suggesting ferroptosis may not represent the dominant programmed mode of cell death in APAP hepatotoxicity [208]. Still, accumulating evidence underscores iron’s broader role in the pathophysiology of hepatotoxicity, with mechanisms that remain to be fully elucidated.

### 6.4 Ischemia-reperfusion injury

IRI represents another form of liver damage, particularly relevant in liver transplantation. There is increasing evidence that ferroptosis significantly contributes to the pathology of hepatic IRI [209], [210]. While ischemia deprives hepatocytes of oxygen and nutrients, ferroptosis predominantly occurs during reperfusion, when restored blood flow provokes a surge in ROS. These ROS, amplified by the Fenton reaction, drive unchecked lipid peroxidation, leading to membrane disruption and the release of damage-associated molecular patterns (DAMPs), which amplify necroinflammatory responses [209], [211]. In line with this, the ferroptosis marker PTGS2 is upregulated during IRI [212], and donor iron overload has been associated with increased risk of graft dysfunction after liver transplantation [210].

A key feature of IRI-induced liver damage is the inactivation of GPX4, leading to unchecked lipid peroxidation and iron accumulation [213]. Pharmacological inhibition of ferroptosis with radical-trapping antioxidants such as liproxstatin-1 or ferrostatin-1 reduces tissue injury, attenuates inflammation, and improves liver function [210], [214]. Beyond synthetic inhibitors, several natural compounds have demonstrated protective effects against hepatic IRI by targeting ferroptotic pathways. Flavonoids such as naringenin suppress ferroptosis by activating the Nrf2/System Xc^−/GPX4 axis, while dihydromyricetin alleviates IRI through inhibition of SPHK1/mTOR signaling [215]. Similarly, galangin reduces reperfusion injury by activating the PI3K/AKT/CREB pathway [216]. Among terpenoids, tanshinone exerts its anti-ferroptotic role by upregulating NQO1, thereby reinforcing antioxidant defense and limiting lipid peroxidation [217]. Collectively, these phytochemicals highlight the therapeutic potential of natural products as adjuncts to conventional ferroptosis inhibitors in the management of IRI.

### 6.5 Hepatocellular carcinoma

HCC is the most common type of primary liver cancer, resulting from a multifactorial interaction between predisposing genetic factors, environmental damage, and metabolic or lifestyle insults [218], [219], [220]. Evasion of programmed cell death is one of the hallmarks of HCC biology that facilitates tumor expansion, cancer development, and chemotherapy and other resistances [221], [222]. Among novel paradigms of regulated cell death, ferroptosis has garnered specific interest. This iron-dependent oxidative damage machinery of ferroptosis independent of apoptosis is triggered via multiple pathways and leads to the accumulation of reactive oxygen species, peroxidation of polyunsaturated fatty acids, and eventual loss of plasma membrane integrity [223], [224], [225]. Induction of ferroptosis has thus emerged as a potential approach to inhibit HCC development; nonetheless, malignant hepatocytes typically develop adaptive protections abrogating ferroptotic signaling or reducing sensitivity to ferroptosis-inducing agents [226], [227], [228]. Within the larger context of controlled cell death, ferroptosis has a particular niche of iron-catalyzed lipid peroxidation and recruitment of certain effector proteins. Within HCC, preclinical models show that pharmacologic or genetic activation of ferroptosis can inhibit tumor growth in vitro as well as in xenografts [229], [230]. Cancer cells tend to develop antioxidant and iron regulation pathways, making them resistant to oxidative lipid damage [231]. Explanation of the molecular mechanism of ferroptotic signaling in HCC is thus critical in an effort to guide rational design for therapy [226]. A number of top-ranking molecular determinants and signaling pathways regulating susceptibility to ferroptosis in HCC is the nuclear factor erythroid 2–related factor 2 (NRF2), a redox-sensitive transcription factor regulating antioxidant and iron homeostasis networks. Hyperactivation of the p62–Keap1–NRF2 pathway allows HCC cells to trigger transcriptionally glutathione metabolism and lipid detoxification genes, giving them robust ferroptotic stress resistance [232], [233]. Therapies based on genetic silencing of NRF2 or pharmacologic inhibition of its activity have been demonstrated to augment the antitumor effect of ferroptosis inducers like sorafenib or erastin [229], [234]. Other regulators, such as YAP/TAZ-dependent transcriptional programs, hypoxia-response elements like HIF-1α–METTL14, and many non-coding RNAs (circRNAs and LncRNAs) that regulate GPX4 or SLC7A11, also influence ferroptotic sensitivity, unveiling the multilayered ferroptosis regulation complexity in HCC [235], [236], [237], [238], [239], [240], [241].

Parallel studies highlight the therapeutic potential of blocking ferroptosis-protective pathways. Agents targeting GSTZ1–NRF2–GPX4 or PI3K–AKT–ABCC5 signaling may be combined with classical inducers to overcome drug resistance. Autophagy-dependent mechanisms, such as ferritinophagy and reticulophagy, also promote lipid peroxidation and iron release, thereby enhancing ferroptosis in resistant tumors [242], [243]. Beyond mechanistic insights, translational research is identifying small molecules including; artesunate, atractylodin, formosanin C, and haloperidol, that induce lipid peroxidation or disrupt iron metabolism, as well as inhibitors of redox defenses (NRF2, G6PD, CISD) to weaken cancer cell resilience [244], [245], [246], [247], [248], [249]. Importantly, ferroptosis intersects with immune surveillance, as genomic and transcriptomic signatures predict prognosis and checkpoint blockade responses in HCC, suggesting ferroptosis competence may shape the tumor immune microenvironment and improve outcomes of ferroptosis–immunotherapy combinations.

Together, the mounting body of evidence places ferroptosis as a potential target in HCC biology [226], [242]. Long-term benefit will likely require multi-modal regimens that simultaneously regulate lipid peroxidation, disrupt iron balance, and overcome anti-ferroptotic defenses. Progress will depend on deeper insight into context-specific signaling, tumor–immune interactions, and predictive biomarkers to enable selective killing of malignant hepatocytes with minimal damage to normal tissue.

### 6.6 Infectious liver disease

About infectious liver disease, in hepatitis C virus (HCV) infection, host lipid desaturation and PUFA-enrichment make a peroxidation-prone milieu that can favor viral replication, and ferroptosis inhibitors have, in some contexts, diminished the activity of direct-acting antivirals, underscoring a nuanced host–virus–lipid triad. In NAFLD/NASH, expansion of peroxidation-susceptible PUFA phospholipids, heightened lipoxygenase and POR/NOX activities, and ER oxidative stress couple steatosis to inflammatory cell death. Targeted ferroptosis blockade in experimental NASH reduces necro-inflammation and fibrosis, suggesting a role for pathway modulation across the steatohepatitis [250], [251], [252], [253]. Notably in malignant hepatocytes frequently rewire to evade ferroptosis, upregulating the SLC7A11–GPX4 axis, deploying FSP1–CoQ10-dependent membrane protection, and shifting membranes toward MUFAs via SCD1 [254]. Nevertheless, this resistance is not absolute. Cytokine signaling like a IFNγ via JAK/STAT can repress system x_c^−, increasing ferroptotic susceptibility; receptor context and iron-storage capacity (ferritin heavy chain) further stratify responses [255], [256]. The variable classification of sorafenib as a ferroptosis inducer likely reflects such context dependence, but combination strategies that constrain antioxidant defenses or re-PUFA-tize tumor membranes are mechanistically sound routes to re-sensitization [257], [258]. HBV and HCV both intersect with ferroptotic mechanisms through their modulation of oxidative stress and iron metabolism. The HBV X protein (HBx) disrupts iron balance by enhancing ROS generation, leading to pathological liver damage [259]. HCV, on the other hand, promotes iron overload via hepcidin suppression, which upregulates duodenal iron transporter-1 and facilitates excess iron uptake [260]. This iron overload worsens hepatic injury and accelerates progression toward HCC by activating ferroptosis-related genes such as FTH1, TFR1, GPX4, and ATF4, particularly in M1-type macrophages [261]. Protective mechanisms have also been described. In HBV, ferroptosis can be mitigated by Nrf2 activation or inhibition of downstream inflammatory pathways including NF-κB, COX-2, STAT, and cyclinD1. Natural products such as curcumin, resveratrol, and silymarin modulate these signaling networks, thereby limiting viral replication and ROS accumulation to reduce ferroptotic cell death [262], [263], [264]. Beyond oxidative stress, HCV alters hepatocyte Kupffer cell signaling and iron uptake to enhance its replication, with TFR1 playing a pivotal role [265]. Fatty acid desaturase 2 (FADS2) has further been identified as a determinant of ferroptosis sensitivity, suggesting that its pharmacological modulation may impair viral propagation [253]. Additional natural agents, including luteolin-7-O-glucoside, ethanolic extracts of mangosteen pericarp, and Pycnogenol, have demonstrated efficacy in reducing HBV/HCV replication and ROS burden, thereby preventing ferroptosis-mediated hepatocyte injury [266], [267]. Overall, diverse phytochemicals, ranging from flavonoids and phenolic compounds to lignans and terpenoids, have shown promise in experimental models of viral hepatitis by acting as ferroptosis inhibitors through antioxidant effects, suppression of inflammatory signaling, or mitochondrial protection [268]. These findings underscore a dual role for ferroptosis in viral hepatitis: as both a driver of HBV/HCV-associated liver injury and a therapeutic target. However, most evidence remains preclinical, and clinical evaluation of ferroptosis-modulating therapies in viral hepatitis is still in its infancy.

### 6.7 Other liver disorder

**Fig. 4:**
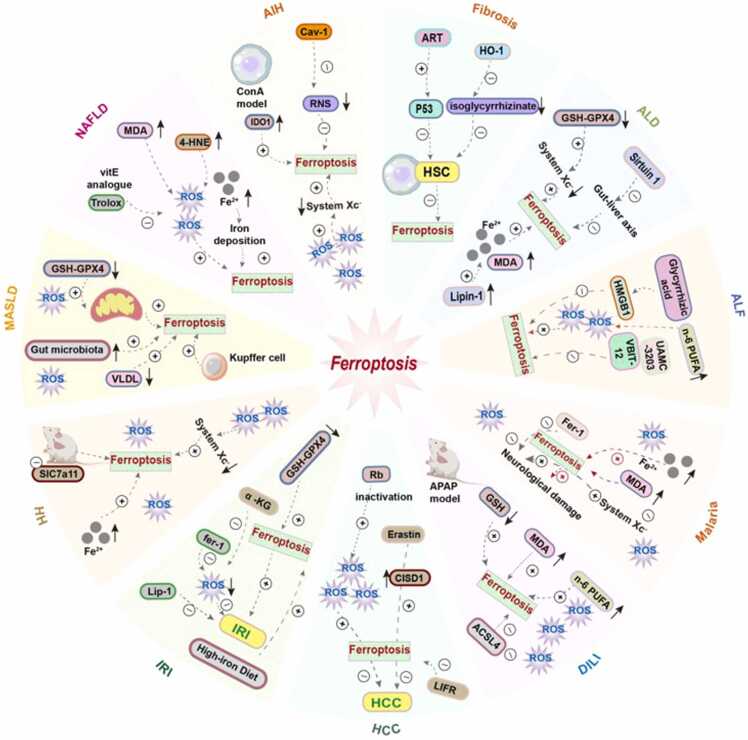
Ferroptosis, a regulated form of iron-dependent lipid peroxidation, underpins a wide spectrum of hepatic disorders. The figure illustrates the mechanistic interplay between ferroptosis and diverse liver pathologies. Each disease engages ferroptotic signaling through distinct yet converging routes, ranging from iron overload and mitochondrial dysfunction to impaired antioxidant systems and dysregulated lipid metabolism, highlighting ferroptosis as a unifying driver of hepatic injury and a promising therapeutic target. Abbreviations: DILI, drug-induced liver injury; ALF, acute liver failure; 4-HNE, 4-hydroxynonenal; MDA, malondialdehyde; VLDL, very Low Density Lipoprotein; Lip-1, liproxstatin-1; Fer-1, ferrostatin-1; α-KG, α-Ketoglutaric acid; CISD1, CDGSH iron-sulfur domain-containing protein 1; ART, artemether; HSC, hematopoietic stem cell; IDO1, indoleamine 2,3-dioxygenase 1; RNS, reactive nitrogen species; HO-1, heme oxygenase-1; HMGB1, high mobility group box 1; ACSL4, acyl-coenzyme synthetase long-chain family member 4; HH, hemochromatosis; HCC, hepatocellular carcinoma; (CC BY-NC-ND 4.0) [14].

Although significant advances were obtained for the involvement of ferroptosis in traditional liver diseases including ALD, NASH, DILI, IRI, and HCC, for others hepatic diseases, the contribution thereof still appears less widely defined. However, evidence grows supporting the implication of ferroptotic signaling in a wider range of inherited, metabolic, congenital, and systemic liver diseases. Classically, Wilson disease arises due to mutations of the ATP7B gene leading to defective excretion of copper into the bile. Secondarily, metabolism of the iron atom has been described as being disrupted in these individuals, with mitochondrial copper-iron crosstalk leading to Fenton chemistry amplification, glutathione depletion, and mitochondrial cristae destruction. Such features parallel ferroptotic vulnerability, and possibly, ferroptosis inhibitors may complement chelating therapy for the prevention of progressive hepatocellular loss [269], [270], [271]. Additionally, in hereditary hemochromatosis, mutations of the HFE or HJV/TFR2 genes increase the labile iron pool, which increases lipid peroxidation and causes mitochondrial accumulation of iron [272], [273]. The liver cell injury demonstrates morphological characteristics of ferroptosis, including compact mitochondria and disrupted membranes. Radical-trapping antioxidants or activation of NRF2 would presumably prevent the onset of cirrhosis and delay hepatocarcinogenesis for individuals with this disorder [273], [274]. α1-Antitrypsin deficiency, primarily considered a protein misfolding disorder, also shows ferroptotic vulnerability via ROS accumulation and ferritinophagy-driven iron release [275]. In sepsis-related liver injury, overwhelming inflammatory storms (IL-6, TNF-α, nitric oxide) override GPX4-dependent antioxidant defenses, while radical-trapping antioxidants such as ferrostatin-1 confer hepatoprotection in experimental models [276], [277]. Autoimmune hepatitis has likewise demonstrated ferroptosis features in concanavalin A–induced models, where iron overload, GSH/GPX4 disruption, and cytokine/RNS signaling sensitize hepatocytes to lipid peroxidation; ferroptosis inhibition may therefore complement immunosuppressive regimens [14], [278]. Finally, infectious diseases such as severe malaria can secondarily induce ferroptosis through hemolysis-driven iron overload and oxidative stress, although this evidence remains preliminary and less directly aligned with hepatotoxicity compared with other conditions [277], [279] (Fig. 4).

## 7 Ferroptosis in response to environmental toxicants

Environmental toxicants, including heavy metals, engineered nanoparticles, and persistent pollutants, are increasingly recognized as potent inducers of ferroptosis in hepatic tissue [280], [281]. Cadmium, Nickel, and Chromium can induce ferroptosis in hepatocytes [282]. These agents converge mechanistically on three hallmarks: iron overload**,** lipid peroxidation**,** and glutathione depletion, driving iron-dependent oxidative cell death [280]. Cadmium disrupts antioxidant homeostasis by downregulating GPX4, SLC7A11, and ferritin, while upregulating ACSL4 and HO-1, enhancing lipid peroxidation and hepatic ferroptosis [283]. Similarly, silver nanoparticles cause mitochondrial damage and increase intracellular Fe²⁺ and malondialdehyde, activating ferroptosis-related pathways, including PPAR and MAPK [284], [285]. Silica nanoparticles promote ferritinophagy and phospholipid hydroperoxide (PL-OOH) accumulation, directly triggering hepatocyte ferroptotic death [286], [287]. These effects are reversible with ferrostatin-1, confirming ferroptotic specificity [287]. Importantly, bioinformatic analyses of exposed liver tissues highlight ferroptosis-linked gene signatures such as Txnip, Egfr, and Arrdc3 associated with metabolic disruption [284]. Moreover, mitochondrial fragmentation, lysosomal iron release, and impaired lipid repair systems have been observed across toxicant models, establishing ferroptosis as a common downstream effector of toxicant-induced liver injury [120], [288]. Therapeutic nanoparticles such as PMOs, and CS-SeNPs have demonstrated protective effects by scavenging ROS, inhibiting ferritinophagy, and restoring GPX4 expression [289], [290]. These findings support the dual role of nanomaterials as both inducers and regulators of ferroptosis, contingent on their physicochemical properties [138], [291].

from nanoparticles and heavy metals, several other environmental pollutants, including pesticides, non-metallic elements, and industrial chemicals, have been identified to induce ferroptosis-mediated liver injury. The herbicide diquat induces oxidative stress, karyolysis, and hepatic cord disorganization in piglets, but is alleviated by natural products such as holly polyphenol extracts (HPE) and glycine, which restore GPX4 at the gene expression level, and HPE further inhibit TFR1-dependent Fe³ ⁺ uptake [124], [125]. Bisphenol A (BPA), an endocrine-disrupting pollutant in plastics, triggers ferroptosis through G protein–coupled estrogen receptor activation, while Artemisia argyi essential oil protects hepatocytes by enhancing GPX4 and lowering intracellular Fe²⁺ [126]. Fluoride exposure likewise culminates in ferroptosis, mediated via the SIRT1/FOXO3 pathway, leading to iron accumulation and lipid peroxidation [128]. Importantly, alpha-lipoic acid, a potent antioxidant, attenuates both fluorosis- and cobalt-induced hepatotoxicity through the System Xc⁻/GPX4 axis, thereby preventing ferroptotic damage [129], [130]. Additionally, diverse pollutants, including ammonia, lead, mercuric chloride, ethyl carbamate, di(2-ethylhexyl) phthalate, aflatoxin B1, and acrylamide, have been documented as potent ferroptosis inducers in liver models, with various natural antioxidants and NACs showing promise in prevention or therapy [131], [132], [133], [134], [135], [136], [137], [138].

All in all, environmental toxicants induce ferroptosis through converging redox and metabolic perturbations. Their effects are biomarker-driven, mechanistically defined, and therapeutically actionable, positioning ferroptosis as a central axis in toxicant-induced hepatotoxicity and a promising target for intervention.

## 8 Targeting ferroptosis by natural compounds

Preventing ferroptosis in liver injury involves complex molecular mechanisms, primarily regulated by the balance of oxidative stress, lipid peroxidation, and iron homeostasis [16], [25], [270]. There is increasing interest in natural products that can modulate these pathways, as they may help both prevent ferroptosis and promote liver recovery [14], [288].

Lipid metabolism is crucial in ferroptosis, as peroxidized PUFAs significantly contribute to cell membrane damage [37], [45]. Enzymes such as ALOX12/15 (lipoxygenases) and ACSL4 (acyl-CoA synthetase long-chain family member 4) play important roles in lipid peroxidation [292], [293]. Several natural compounds have been identified that can modulate their activity to prevent ferroptosis; Ginsenoside Rd, genipin, and bicyclol have been shown to reduce lipid peroxidation and oxidative stress by inhibiting these enzymes' activity [158], [294]. Additionally, Baicalein, a flavonoid, also exhibits promising effects in inhibiting ferroptosis by decreasing the activity of ACSL4 and ALOX12/15, thus protecting against oxidative damage in liver injury models [295], [296], [297]. Vitamin E and Trolox, diminish lipoxygenase action and neutralize hydroxyl radicals [298]; coenzyme Q10 (CoQ10) acts as a lipophilic radical-trapping antioxidant inhibiting the spread of lipid peroxides; selenium and sodium selenite aid in the restoration of GPX4 protein [299]; β-mercaptoethanol (β-ME) accelerates cystine reduction to cysteine and thus outflanks system Xc− inhibition; and SRS11–92 lessens lipid hydroperoxide build-up through inhibition of lipoxygenases [300].

Additionally, the NRF2 (Nuclear Factor Erythroid 2–Related Factor 2) pathway regulates the antioxidant response, especially regarding ferroptosis [301]. Activation of NRF2 upregulates several antioxidative genes, including GPX4, which is essential for detoxifying lipid peroxides; Silibinin and genistein activate the NRF2/HO-1 pathway, offering hepatoprotection by reducing oxidative stress and limiting ferroptosis [16], [302], [303], [304]. In models of iron overload, epigallocatechin-3-gallate (EGCG) activates NRF2, enhancing antioxidant defenses in the liver by increasing GPX4 levels and decreasing lipid peroxidation [305].

Notably, mitochondria play a dual role in ferroptosis: while they generate ROS as part of their metabolic activities, they can also serve as targets for cellular defence mechanisms against lipid peroxidation [306], [307]. Maintaining mitochondrial health is critical for preventing ferroptosis; PINK1/Parkin-mediated mitophagy is a mechanism that prevents mitochondrial damage, reduces ROS production, and thus inhibits ferroptosis [308], [309]. Silibinin promotes mitophagy by interacting with the PINK1/Parkin pathway, which helps eliminate defective mitochondria and decrease ROS accumulation in liver cells [310], [311]. Notably, Ginsenoside Rd also protects liver cells by modulating mitochondrial function and preventing excessive ROS production, which helps to prevent ferroptosis under stress conditions [312], [313].

In addition, iron dysregulation is a hallmark of ferroptosis, with excessive iron accumulation being a key driver of lipid peroxidation [120], [314]. Several natural compounds can help modulate iron levels and reduce iron-mediated oxidative stress; EGCG has demonstrated the ability to decrease the intestinal absorption of non-heme iron and enhance the expression of ferritin (FTH1 and FTL), which helps prevent hepatic iron overload [315]. Through its antioxidant and anti-inflammatory properties, Taurine increases the expression of SLC7A11 and GPX4, promoting iron homeostasis and inhibiting ferroptosis in liver injury models [316], [317]. Nevertheless, drugs such as Fuzheng Yanggan, a traditional herbal medicine, inhibit ferroptosis via SLC7A11 and GPX4 stabilization, while ferrostatin [318].

All in all, research into the role of ferroptosis in liver injury is expanding. Targeting ferroptosis with natural products offers a promising approach to developing therapeutic strategies for liver diseases [117]. Evidence suggests that natural compounds such as ginsenoside Rd, silibinin, and EGCG can inhibit ferroptosis, highlighting their potential as adjunct therapies for conditions such as liver ischemia-reperfusion injury, alcoholic liver disease, and DILI [319], [320]. However, further exploration is needed to understand these natural compounds' precise mechanisms of action and their potential clinical applications. More studies are required to validate their efficacy in clinical settings, establish optimal dosages, and assess potential side effects or drug interactions. Importantly, combining ferroptosis inhibitors with other therapies may represent a comprehensive approach to treating liver diseases involving oxidative stress and iron dysregulation [14], [319], [321], [322].

Further natural compounds with distinct mechanisms have also demonstrated the ability to modulate ferroptosis and protect against liver injury. Fucoidan, a marine polysaccharide, mitigates alcohol-induced hepatic ferroptosis by restoring redox balance, regulating iron homeostasis, and activating the p62/Nrf2/SLC7A11/GPX4 axis. Melatonin offers circadian-linked protection against ethanol-induced liver damage through BMAL1-dependent activation of the Nrf2-ARE pathway and downstream anti-ferroptotic targets such as HO-1, FPN, and SLC7A11 [323]. Additionally, natural compounds like astaxanthin (ASX) and retinoic acid (RA) also demonstrate hepatoprotective effects [324], [325]. RA reduces LPS-induced hepatic iron overload and ferroptosis via RARβ-mediated activation of Nrf2/HO-1/GPX4 and iron export proteins FTL/FPN [326]. Lastly, maresin-1 and its conjugate MCTR1 exhibit potent anti-ferroptotic and anti-inflammatory effects by activating the Nrf2–SLC7A11–GPX4 axis [327], [328]. These compounds reduce liver damage markers and oxidative stress in both sepsis and ischemia-reperfusion models, further supporting their therapeutic potential.

## 9 Targeting ferroptosis by synthetic small molecules

The targeted modulation of ferroptosis by synthetic small molecules represents an essential strategy for both mechanistic dissection and therapeutic intervention in hepatotoxicity and related liver disorders. These molecules are themselves specific biochemical probes that induce ferroptotic activities or confer protection against lipid peroxidation and iron homeostasis. By determining their modes of action, researchers have uncovered mechanisms through which small molecules modify the balance of cellular survival and ferroptotic death of hepatocytes and modulate resulting tissue outcomes.

Ferroptosis inducers can generally be segregated into mechanistic classes. Class I inducers mostly act upon system Xc− and thus limit cystine uptake and consume intracellular glutathione. Erastin, sorafenib, sulfasalazine, and extracellular glutamate illustrate this class through the limitation of antioxidant protection and sensitization of the hepatocyte to oxidative damage [232], [300], [329], [330]. Class II inducers, conversely, act directly upon GPX4, the key enzyme involved in detoxifying lipid hydroperoxides. RSL3 and ML-162 are established prototypic members that each covalently inhibit GPX4 and induce unbridled lipid peroxide accrual [331], [332], [333]. Far beyond these traditional classifications come other ferroptosis inducers such as FIN56, decreasing GPX4 levels while binding to squalene synthase and inhibiting coenzyme Q10 formation [334]; dihydroartemisinin (DHA), decreasing GPX4 expression [335]; ferric ammonium citrate (FAC), inducing an iron overload and promoting lipid peroxidation [300]; and l-buthionine-(S,R)-sulfoximine (BSO), inhibiting the glutamate-cysteine ligase and consuming pools of GSH [336]. Other molecules displaying pro-ferroptotic effects are; FINO [2], indirectly inhibiting GPX4 and forming lipid peroxides through a direct oxidation of iron; and brequinar, a dihydroorotate dehydrogenase (DHODH) inhibitor that inhibits ubiquinone reduction and disrupts redox buffer capacity [137], [337].

Accompanying these discoveries are inhibitors of ferroptosis, used to protect hepatocytes from ferroptotic failure during models of hepatotoxicity. Ferrostatin-1 and liproxstatin-1 are typical radical-trapping antioxidants that efficiently scavenge lipid radicals and halt lipid peroxide propagation [338], [339]. Deferoxamine (DFO) and ciclopirox olamine (CPX) prevent ferroptosis through the action of iron chelation and thus limit iron-driven Fenton reactions [300]. Others still are inhibitors that include baicalein as a powerful lipoxygenase activity suppressing agent; XJB-5–131 as an antioxidant per lipid peroxidation inhibitor [340]; and gliptin-class molecules, such as vildagliptin, alogliptin, and linagliptin, as inhibitors of DPP4 in order to prevent lipid peroxidation mediated through DPP4 [341]. Notably, VBIT-12, a VDAC1 oligomerization inhibitor, protects against APAP-induced ferroptosis by preserving mitochondrial function and lipid stability [342], [343]. Steroid derivative 17α-hydroxypregnenolone (17-OH PREG) alleviates ferroptosis by stimulating GPR56, which promotes CD36 degradation and reduces PUFA-containing phospholipids [169], [344].

Evidence from models of hepatotoxicity presents the complex functions of the molecules. For example, 3,5,5-trimethyl-hexanoyl ferrocene (TMHF) and artemether (ART) induce ferroptosis via mitochondrial and SLC7A11/GPX4 mechanisms [345], [346], respectively, and haloperidol induce ferroptotic injury via HO-1 induction and inhibition of GPX4 [144], [347]. Both natural and chemical molecules, such as trigonelline [348] and AICAR [349], induce Nrf2 signaling mechanisms to induce thethresholds of ferroptosis.

Altogether, the breadth of synthetic and semi-synthetic small molecules, including erastin, sorafenib, sulfasalazine, RSL3, ML-162, FIN56, FINO [2], brequinar, ferrostatin-1, liproxstatin-1, CPX, SRS11–92, XJB-5–131, gliptins (vildagliptin, alogliptin, linagliptin), TMHF, haloperidol, AICAR, BSO, DHA, ART, MI (magnesium isoglycyrrhizinate); represents the vastly diversified pharmacological resources accessible to the exploration and therapeutic manipulation of ferroptosis. By systematically stratifying these compounds according to their molecular targets and pharmacodynamic effects, future studies may refine hepatoprotective strategies and advance ferroptosis-centered therapies for drug-induced, metabolic, and inflammatory liver diseases.

## 10 Targeting ferroptosis by nanoparticles

Nanoparticles (NPs) have shown dual potential in inducing liver injury and serving as therapeutic agents for liver-related conditions [350]. Among them, silica nanoparticles (SiNPs), widely produced and utilized synthetic nanomaterials, have attracted increasing concern due to their implications for environmental health and safety (EHS). Although the mechanisms remain unclear, SiNPs have been implicated in causing liver damage [287]. In in vivo studies, SiNP exposure led to histopathological liver injury accompanied by ferroptosis. Key features included ferritinophagy and lipid peroxidation [288], [351]. In vitro experiments using L-02 cells revealed decreased cell viability, rupture of mitochondrial membranes and cristae, accumulation of intracellular and mitochondrial ferrous iron, elevated PL-OOH, and impaired lipid peroxidation repair, hallmarks consistent with ferroptosis induction. Importantly, ferrostatin-1, a known ferroptosis inhibitor, was able to suppress SiNP-induced lipid peroxidation and cytotoxicity [352], [353]. In a recent study by Shan et al. (2023), ultrasmall poly(acrylic acid)-coated Mn₃O₄ nanoparticles (PAA@Mn₃O₄-NPs, abbreviated as PMOs) were synthesized via a simple one-pot method [291]. These nanoparticles exhibit reactive oxygen species scavenging and mimic multiple antioxidant enzymes [354]. Mechanistically, following macropinocytic uptake, PMOs predominantly localize in lysosomes, where they suppress ferroptosis by detoxifying ROS, inhibiting ferritinophagy-mediated iron release, and sustaining mTOR signaling [291]. In rodent models of acetaminophen- and ischemia/reperfusion-induced liver injury, PMO treatment significantly reduced lipid peroxidation and liver damage. Further supporting evidence comes from integrative bioinformatic analyses [291], [355]. One study combined ferroptosis-related gene datasets with the GEO dataset GSE139560, which includes liver tissues from rodents exposed to silver nanoparticles (AgNPs) [284]. This intersectional analysis identified three ferroptosis-associated genes: Arrdc3, Txnip, and Egfr. Cross-referencing with disease models from datasets GSE111407 and GSE183158 revealed a strong association between AgNP exposure, insulin signaling disruption, and glucose metabolism, linking them to the aforementioned genes [356], [357]. AgNP exposure also activated ferroptosis, associated pathways such as PPAR and MAPK [284]. These findings were experimentally validated in zebrafish models, where AgNPs increased Fe²⁺ and MDA levels and caused marked mitochondrial abnormalities in liver tissue, further confirming the role of ferroptosis in AgNP-induced hepatotoxicity and metabolic dysfunction [358], [359]. Notably, ASX activates the Nrf2/HO-1 pathway and enhances autophagy, especially when delivered via liver-targeted nanoparticles (HMSN@ASX) [360]. In addition, cadmium, a well-documented environmental toxicant, can severely damage the liver among other organs [361]. Animal studies have demonstrated that chitosan-coated selenium nanoparticles (CS-SeNPs) effectively counteract Cd-induced hepatotoxicity [290], [362]. Exposure to CdCl₂ led to elevated liver index, increased MDA, ALT, and AST, and disrupted antioxidant balance, including serum GSH-Px activity [363]. CdCl₂ also downregulated hepatic ferritin, TfR1, and GPX4, while upregulating ACSL4 and HO-1 key players in ferroptosis [364], [365]. CS-SeNP treatment successfully reversed these biochemical and histological changes, notably restoring GPX4 expression and mitigating Cd-induced liver injury [366], [367]. Despite NPs therapeutic promise, nanoparticle-based systems raise concerns regarding long-term bioaccumulation, off-target toxicity, and immunogenicity. Further preclinical and clinical studies are required to establish their safety profile and optimize pharmacokinetic behavior before translation into human applications **(**Table 1**).**

**Table 1:** Overview of pharmacological agents, natural compounds, and nanotechnology-based strategies modulating ferroptosis in experimental and clinical models of liver disease.

**Classification**	**Name**	**Composition / Nature**	**Targeted pathway**	**Study type**	**Cell lines / Animal models**	**Mechanism**	**Ref.**
Natural agents	Corosolic acid (CA)	Natural pentacyclic triterpene acid	HERPUD1–MDM2–GSS axis; GSH synthesis/antioxidant defense	In vivo, in vitro	Bel−7402, Bel−7404, HepG2, SMMC−7721, SK-Hep1, HL−7702; Athymic nude mice (WT/KO Bel−7402 xenografts; CA 10 mg/kg, IKE 50 mg/kg, combo)	CA upregulates HERPUD1 → decreases MDM2 ubiquitination (stabilizes MDM2) → MDM2 ubiquitinates GSS → ↓GSS protein → ↓GSH synthesis → heightened ferroptosis sensitivity. CA pretreatment augments erastin/RSL3-induced lipid ROS and cell death (rescued by Fer−1); Fe²⁺ unchanged with CA. In xenografts, CA±IKE lowers tumor growth and GSH; HERPUD1 knockout blunts effects and reduces GSS ubiquitination.	[368]
Natural agents	Solasonine	Natural glycoalkaloid from Solanum melongena	GPX4 / GSH redox system (↓GPX4, ↓GSS → GSH depletion)	In vivo, in vitro	HepG2, HepRG; BALB/c nude mice (HepG2 xenograft; solasonine 50 mg/kg)	Solasonine downregulates GPX4 and GSS, depleting GSH and elevating lipid ROS → ferroptosis; cell death reversed by ferrostatin−1 and deferoxamine; suppresses tumor volume/weight and migration/invasion in models.	[369]
Synthetic small molecules	Sophoridine derivative 6j	Small-molecule derivative of sophoridine	ER-stress → PERK/ATF4 → ATF3 axis; ferroptosis markers (Fe²⁺/ROS/MDA)	In vivo, in vitro	HepG2	6j induces ferroptosis: ↑intracellular Fe²⁺, ROS, MDA; effects rescued by Fer−1. 6j upregulates ATF3 via ER-stress (blocked by 4-PBA); ATF3 knockdown reduces Fe²⁺/ROS/MDA increases and restores viability. In vivo: tumors ↓, ATF3↑, Fe²⁺/MDA↑, Ki−67↓.	[370]
Natural agents	Scutellaria barbata (herbal extract)	Traditional Chinese Medicine herb extract	Iron & lipid-ROS axis; xCT/GPX4 pathway: ↑Fe²⁺, ↓mitochondrial membrane potential; ↓SLC7A11, ↓GPX4; ↑IREB2, ↑ACSL4 → lipid peroxidation & ferroptosis	In vivo, in vitro	SMMC−7721, HepG2, Huh7; Nude mice (HepG2/Huh7 xenografts)	Induces ferroptosis by promoting iron peroxidation and lipid ROS metabolism; elevates tumor iron and suppresses SLC7A11 in vivo → reduced tumorigenicity	[371]
Natural agents	Heteronemin	Marine terpenoid (from Hippospongia sp.)	ROS → MAPK (↑JNK/↑p38, ↓ERK); GPX4 axis (ferroptosis)	In vivo, in vitro	HA22T/VGH, HA59T/VGH	↑ROS; SOD2↑/SOD1↓; apoptosis: caspase cascade, Bax↑, PARP−1 cleavage; ferroptosis: GPX4↓, lipid peroxidation-dependent death; JNK/p38 inhibitors attenuate killing; ERK downregulated, dual apoptosis/ferroptosis inducer	[372]
Natural agents	Gallic acid (GA)	Plant-derived phenolic acid (trihydroxybenzoic acid)	Wnt/β-catenin signaling inhibition → downregulation of SLC7A11 & GPX4	In vitro	HepG2	Blocks β-catenin nuclear transport → inactivates Wnt/β-catenin → decreases GPX4 & SLC7A11 → lipid ROS accumulation → ferroptosis induction	[373]
Natural agents	Formosanin C (FC)	Diosgenin-type saponin from Paris formosana	Ferritinophagy → ferroptosis (NCOA4-mediated ferritin degradation)	In vitro	HepG2, Hep3B	FC induces lipid ROS and autophagic flux (↑LC3-II; Baf-sensitive), promotes NCOA4–FTH1 colocalization and ferritin degradation (EM immunogold), ↑TFRC (iron uptake), ferroptosis reversed by Ferrostatin−1; effect stronger in HepG2 (high NCOA4/low FTH1; FTH1 negatively correlates with ferroptosis sensitivity). Potential against apoptosis-resistant HCC.	[247]
Natural agents	SSPH I	Steroidal saponin from Schizocapsa plantaginea	Ferroptosis via iron-metabolism dysregulation (iron overload → lipid peroxidation)	In vitro	HepG2	Anti-proliferative/anti-migration; induces ROS↑, MDA↑, GSH↓, ferroptotic mito changes; effects rescued by Ferrostatin−1 or ciclopirox. xCT unchanged; SLC7A5↑; TFR1↑ and Fpn↑ with net Fe²⁺ accumulation; also triggers apoptosis and G2/M arrest.	[374]
Natural agents	Saikosaponin A (SsA)	Bioactive triterpenoid saponin from Radix Bupleuri	Glutathione metabolism / ER stress–ATF3 axis	In vivo, in vitro	HepG2, Huh7; Nude mice (HepG2/Huh7 xenografts)	↓Cell viability (dose/time-dependent); induces ferroptosis via ER stress → ATF3↑ → suppression of SLC7A11 → GSH↓, lipid peroxidation↑, Fe²⁺ accumulation↑. Rescue by Fer−1, DFO, or GSH (not by Z-VAD-FMK). Confirms ferroptosis not apoptosis.	[375]
Synthetic small molecules	Entospletinib	Small-molecule drug (SYK inhibitor; anti-ferroptosis activity independent of SYK)	Ferroptosis (↓ lipid peroxidation/mito-ROS; ↓ HMOX1; ↑ SLC7A11/GPX4)	In vivo, in vitro	A375, H1299, HK−2, 786-O cells; C57BL/6 mice (ConA-induced ALI; cisplatin-induced AKI)	Blocks mitochondrial ROS and lipid peroxidation; suppresses RSL3-induced HMOX1; restores SLC7A11/GPX4; protects from ferroptosis across multiple inducers	[376]
Synthetic small molecules	VBIT−12	VDAC1 oligomerization inhibitor	Mitochondrial VDAC1 oligomerization; MMP/OCR; lipid peroxidation; mitochondrial Fe²⁺	In vivo, in vitro	PMHs, Hepa1–6, human DILI biopsies	Inhibits VDAC1 oligomerization → preserves MMP & OCR, ↓ mito-Fe²⁺/ROS/4-HNE; restores cardiolipin & ceramide (↑TAZ, ↑SMPD1); no effect on CYP2E1	[20], [376]
Natural agents	Fucoidan	Natural brown-algae polysaccharide (α-L-fucose sulfate; ∼220–260 kDa)	p62–Keap1–Nrf2–SLC7A11/GPX4; hepatic hepcidin–intestinal DMT1/FPN1; iron homeostasis	In vivo	Sprague–Dawley rats (chronic alcohol exposure, 16 weeks)	↓ ROS/MDA; ↑ GSH/GPX; ↓ ferritin & hepatic iron deposition; restores hepcidin; ↓ DMT1/FPN1; activates p62–Keap1–Nrf2 → ↑ SLC7A11 & GPX4; inhibits alcohol-induced ferroptosis	[169]
Natural agents	17-α-Hydroxypregnenolone (17-OH PREG)	Endogenous steroid; agonist of GPR56/ADGRG1	GPR56–CD36 axis; PUFA-phospholipid turnover; ferroptosis defense	In vivo, in vitro	Primary hepatocytes; Mice (DOX-induced liver injury; ischemia–reperfusion injury)	Activates GPR56 → promotes CD36 degradation via endocytosis-lysosomal pathway → ↓ PUFA-phospholipids → resistance to ferroptosis; protects liver before/after injury; mutants of GPR56 lose response	[344]
Regulator	HUWE1/MULE (HECT E3)	Ubiquitin E3 ligase (HECT domain)	TfR1 ubiquitination → proteasomal degradation; iron uptake/homeostasis; ferroptosis	In vivo, in vitro	Primary hepatocytes; Hepa−1c1c7, Huh7; Hepatocyte-specific Huwe1 HKO mice (I/R, CCl₄); Human liver transplant donor/recipient biopsies	HUWE1 binds/ubiquitinates TfR1 (K48-linked) → ↓ TfR1 stability → ↓ labile iron & lipid peroxidation; Huwe1 loss ↑ Acsl4, 4-HNE, MDA; Ferrostatin−1 or TfR1 inhibition (Ferristatin II / shRNA) rescues cells and mice	[377]
Natural agents	Melatonin (MLT)	Endogenous indoleamine (N-acetyl−5-methoxytryptamine)	Nrf2/HO−1 axis; GPX4/SLC7A11; pAMPK–SREBP1c–PPARα/γ; mitochondrial ROS/iron metabolism	In vivo, in vitro	HepG2 (FFA-induced NAFLD); C57BL/6 mice (HFD-induced NAFLD, ±MLT 10–20 mg/kg)	↑ GSH/SOD; ↓ ROS, MDA, iron deposition; restores GPX4 & SLC7A11; activates Nrf2/HO−1; ↓ Keap1; reduces lipogenesis via ↑pAMPK & PPARα, ↓PPARγ/SREBP1c/FAS; protects histology & physiology in NAFLD	[378], [379]
Natural agents	Fibroblast Growth Factor 21 (FGF21)	Endocrine FGF family hormone (mainly hepatic)	HO−1 ubiquitination/degradation; NRF2 activation; GPX4/SLC7A11; iron metabolism (FTH/L, FPN, TFR1)	In vivo, in vitro	Primary hepatocytes (FAC-induced ferroptosis); C57BL/6J mice (iron dextran overload; FGF21 overexpression/knockdown; recombinant protein); Liver fibrosis model	FGF21 ↑ under iron overload; recombinant/overexpression ↓ LIP, ROS, MDA; ↑ GSH; restores NRF2, GPX4, SLC7A11, FTH/L, FPN; inhibits HO−1 via ubiquitination → proteasomal degradation; protects against iron overload-induced ferroptosis, liver injury, fibrosis; knockdown worsens injury	[380], [381]
Synthetic small molecules	Retinoic Acid (RA)	Active vitamin A metabolite (all-trans RA); RAR agonist	Nrf2/HO−1/GPX4; RARβ/γ signaling; iron metabolism (FTH/L, FPN)	In vivo, in vitro	Primary hepatocytes; C57BL/6J mice (LPS-induced acute liver injury, ±RA 15 mg/kg)	RA ↓ serum ALT/AST, iron, non-heme iron, LIP; ↑ FTH/L & FPN; ↓ ROS/MDA; restores GSH; ↑ HO−1, GPX4, Nrf2 activity; inhibits LPS-, RSL3-, and erastin-induced ferroptosis; RARβ knockdown abolishes protection → anti-ferroptotic effect partly via RARβ	[20], [382]
Natural agents	Maresin−1 (MaR1)	Specialized pro-resolving mediator (SPM) derived from DHA	Nrf2/SLC7A11/GPX4 axis; Keap1 suppression; anti-inflammatory cytokine modulation	In vivo, in vitro	AML12 hepatocytes; C57BL/6J mice (CLP-induced sepsis model, ±MaR1 100 ng/mouse)	MaR1 ↓ ALT/AST, ROS, MDA, iron; ↑ GSH & GSH/GSSG ratio; restores GPX4 & SLC7A11; activates Nrf2 (↑ nuclear translocation, ↓ Keap1); reduces TNF-α/IL−6; protection abrogated by ML385 or Nrf2 knockdown → anti-ferroptotic & anti-inflammatory	[22]
Natural agents	Dehydroabietic acid (DA)	Plant-derived diterpenoid resin acid	Keap1/Nrf2–ARE pathway (↑ HO−1, GPX4, GSH, FSP1)	In vivo, in vitro	Hepatocyte models; Mice (HFD-induced NAFLD)	DA binds Keap1 (VAL512 & LEU557H-bonds) → releases Nrf2 → activates ARE → ↑ antioxidant defenses (HO−1, GPX4, GSH, FSP1) → ↓ ROS, MDA, lipid peroxidation → inhibits ferroptosis and ameliorates NAFLD	[383]
Natural agents	Ginkgolide B (GB)	Major terpene lactone from Ginkgo biloba	PXR activation (↑ CYP3A11, ↑ MRP3; ↓ SREBP−1, ACC, FAS, SCD1)	In vivo, in vitro	C57BL/6 mice (HFD-induced obese/NAFLD model)	Selective hPXR agonist: reduces body weight and serum TG; improves hepatic steatosis; lowers serum total bile acids; ↑ hepatic GSH; increases fecal TG (suggesting ↓ intestinal TG absorption); modulates bile-acid/lipid metabolism via PXR signaling in liver & intestine	[384]
Natural agents	Green tea / Epigallocatechin gallate (EGCG)	Tea catechin; potent antioxidant from Camellia sinensis	NRF2, AMPK, SIRT1, NF-κB, TLR4/MYD88, TGF-β/SMAD, PI3K/Akt/FoxO1	In vivo, in vitro	Hepatocyte and hepatic stellate cell lines; Rodent models (HFD/NASH); Human NAFLD/NASH trials	Alleviates oxidative stress (↑NRF2/ARE, ↑GSH, ↓MDA/ROS), improves lipid metabolism (↑AMPK/SIRT1 → ↓SREBP−1c/ACC/FAS), anti-inflammatory (↓NF-κB; blocks TLR4/MYD88 signaling), anti-fibrotic (↓TGF-β/SMAD; affects PI3K/Akt/FoxO1 in HSCs), and shows chemopreventive signals vs NAFLD-related HCC; clinical data show improvements in ALT/AST, TG/TC/LDL-C, insulin resistance, and hepatic fat. Note: very high EGCG doses may cause GI side effects—moderate dosing favored.	[305]
Natural agents	Dihydroartemisinin (DHA)	Semi-synthetic artemisinin derivative	Autophagy–NCOA4–ferritinophagy axis; ferroptosis markers (ROS ↑, lipid peroxidation ↑, GSH ↓, Fe²⁺ overload)	In vivo, in vitro	Activated hepatic stellate cells; Mouse hepatic fibrosis model	DHA activates autophagy and ferritinophagy via ATG5–NCOA4 axis → induces ferroptosis in HSCs → reduces ECM deposition → alleviates hepatic fibrosis. Ferroptosis inhibitors (Fer−1, Lip−1) block effect.	[385]
Natural agents	Artesunate	Artemisinin hemisuccinate derivative (water-soluble)	Ferritinophagy-mediated ferroptosis in HSCs; autophagy genes ↑ (LC3, Atg3/5/6/12), p62 ↓; FTH1 & NCOA4 ↓; ferroptosis markers: Fe²⁺ ↑, lipid ROS/LPO/MDA ↑, GSH & GPX4/NADPH ↓	In vivo, in vitro	Primary mouse hepatic stellate cells; Human HSC line LX−2; Mice (CCl₄-induced liver fibrosis model)	Artesunate induces ferritinophagy → increases labile iron → lipid peroxidation → ferroptotic death of activated HSCs → antifibrotic effect. Ferroptosis blockade (DFO) abolishes benefit; ferritinophagy inhibition (chloroquine) impairs ferroptosis and antifibrosis.	[144]
Natural agents	Oat phenolic compounds (OPC)	Polyphenolic fraction from oat bran	Gut microbiota modulation; Anti-oxidative stress pathways	In vivo	C57BL/6J mice (HFD-induced NAFLD/NASH model)	Attenuates metabolic syndrome: ↓ body weight, TG, TC, LDL-C; ↑ antioxidant enzymes (SOD, GSH-Px, T-AOC); ↓ MDA. Restores gut microbiota composition (↑ Bacteroidetes, ↓ Firmicutes, ↑ Eubacterium, ↓ Alistipes & Lachnospiraceae). Improves glycolipid metabolism & reduces hepatic lipid accumulation.	[386]
Natural agents	Acacetin	Natural flavone (di-hydroxy, mono-methoxy)	ER-stress → ferroptosis axis (ER stress upstream of ferroptosis)	In vivo, in vitro	HepG2 (OA+LPS); Mice (HFD-induced NAFLD, 10–50 mg/kg/day)	Lowers body weight gain and serum TC/TG/LDL/AST/ALT; reduces hepatic lipid accumulation, iron overload & lipid peroxidation; downregulates lipogenesis genes; inhibits ER stress and ferroptosis. Erastin or tunicamycin abolish benefits; GSK2606414 (ER-stress inhibitor) and Fer−1 define ER stress as upstream of ferroptosis.	[387]
Synthetic small molecules	Ferrostatin−1 (Fer−1)	Synthetic small-molecule radical-trapping antioxidant (ferroptosis inhibitor)	SLC7A11/xCT–GPX4 axis; TFRC/TFR1–mediated iron overload; ROS/lipid peroxidation; mitochondrial injury	In vivo	Sprague–Dawley rats (ricin-induced liver injury; Fer−1 pretreatment)	Fer−1 pretreatment ↑ GSH, ↑ GPX4 & SLC7A11; ↓ iron (Prussian blue/assay), ↓ MDA, ↓ ROS, ↓ TFR1; ameliorated mitochondrial shrinkage & membrane rupture (TEM); reduced ALT/AST and hepatic necrosis; prolonged survival though not preventing lethality	[388]
Synthetic small molecules	Liproxstatin−1 (LPT1)	Synthetic radical-trapping antioxidant	Ferroptosis (ACSL4/ALOX15, GPX4); PANoptosis (apoptosis, pyroptosis, necroptosis)	In vivo, in vitro	AML12; C57BL/6J mice (HFHF-induced MAFLD; MCD diet–induced NASH)	↓ lipid peroxidation (MDA, 4-HNE); restores GSH & GPX4; ↓ ACSL4/ALOX15; improves steatosis, insulin resistance & fibrosis; blocks apoptosis (Bax↓, Bcl-xL↑), necroptosis (p-MLKL↓), pyroptosis (caspase−1, GSDMD↓); inhibits PANoptosis (caspase−6/−8 cleavage)	[389], [390]
Synthetic small molecules	Deferoxamine (DFO)	Iron chelator (FDA-approved drug)	Ferroptosis (iron overload, lipid peroxidation)	In vivo	C57BL/6J mice with high-fat/high-fructose diet (MAFLD model)	Chelates excess hepatic iron → ↓ non-heme iron & ferritin; ↓ MDA (lipid peroxidation); ↓ inflammatory cytokines (IL−6, IL−1β, TNFα); ↓ fibrotic markers (Col1α1, Col3α1, TGFβ); ↓ AIF without affecting GPX4; protects against steatosis, inflammation, and fibrosis	[391]
Natural agents	Vitamin A (Retinol / Retinal / Retinoic Acid; carotenoids)	Fat-soluble vitamin	RAR/RXR; NRF2; lipid metabolism (SCD/ACSL3)	In vivo, in vitro	Primary hepatocytes; Liver injury models (LPS-induced ALI)	Upregulates Gpx4, Fsp1, Gch1 via RAR/RXR; increases MUFA synthesis via SCD → reduces lipid peroxidation; promotes NRF2 nuclear translocation by carotenoids; protects against RSL3/erastin-induced ferroptosis	[392]
Natural agents	Vitamin C (Ascorbate / Dehydroascorbic acid)	Water-soluble vitamin	SLC7A11/GPX4; AMPK/NRF2→HMOX1; ferritinophagy	In vivo, in vitro	Mouse hepatocytes; Liver model under PD−1 immunotherapy	Low dose: ↑SLC7A11 & GPX4 → anti-ferroptotic in hepatocytes; High dose: induces H₂O₂ production, iron release via ferritinophagy → may trigger ferroptosis (mainly in tumor cells, less in normal liver)	[392]
Natural agents	Vitamin E (Tocopherols / Tocotrienols)	Lipophilic antioxidant (radical-trapping)	Lipid peroxidation chain reaction; ALOX inhibition	In vivo, in vitro	NAFLD/NASH models; APAP-induced acute liver failure model	Scavenges lipid radicals; protects GPX4-deficient T cells; reduces ALT/AST, steatosis and inflammation in NAFLD/NASH; prevents APAP-induced acute liver damage	[392]
Natural agents	Vitamin K (K1, K2; VKH₂)	Fat-soluble quinone vitamin	FSP1/VKORC1L1 cycle → VKH₂ (radical-trapping antioxidant)	In vivo, in vitro	Gpx4-knockout liver injury models	VKH₂ traps lipid radicals directly; VKORC1L1/FSP1 enzymatic cycle regenerates VKH₂; reduces ferroptotic liver injury in Gpx4-deficient mice	[392]
Synthetic small molecules	N-acetyl-L-cysteine (NAC) / N-acetyl-D-cysteine (D-NAC)	Small-molecule antioxidant; cysteine prodrug (L-NAC) and non-metabolizable enantiomer (D-NAC)	GPX4 enzymatic activity (direct reducing substrate); cysteine replenishment	In vitro	Multiple hepatic cell models (ferroptosis induction assays)	NAC replenishes cysteine → supports GSH synthesis; both L-NAC and D-NAC act as direct reducing substrates for GPX4 (independent of system Xc⁻ or FSP1); counteract lipid peroxidation; identifies β-mercaptoethanol and other thiols as GPX4 substrates; broad anti-ferroptotic protection	[393]
Nanomaterial	GOA/miR−363–5pi nanocomplex; GOA/miR−765i nanocomplex	Gemcitabine–oleic acid (GOA) prodrug nanoparticles delivering miR−363–5p/miR−765 inhibitors	↑LHPP (histidine phosphatase) → ↓PI3K/Akt phosphorylation → ↓GPX4 → lipid peroxidation → ferroptosis; ICD activation	In vivo, in vitro	HepG2, SMMC−7721, Bel−7402; BALB/c nude mice (Bel−7402 xenografts)	Efficient miRNA encapsulation (≈200 nm, −20 mV); enhances LHPP expression, inactivates PI3K/Akt, downregulates GPX4, elevates lipid peroxides; Fer−1 rescues cell death; induces immunogenic cell death (↑CRT, ↑ATP, ↑HMGB1); tumor inhibition ≈ 88–89 % without systemic toxicity.	[394]
Natural agents	(20S)-Protopanaxatriol ((20S)-APPT)	Ginsenoside derivative (from Panax ginseng)	FSP1 inhibition; ACSL4-dependent arachidonic acid oxidation; mitochondrial ROS	In vivo, in vitro	HCC cell lines; CCA cell lines; Xenograft mouse models	Directly targets plasma membrane FSP1 → ↓ CoQ10 reduction capacity → ↑ ACSL4-mediated PUFA oxidation & mitochondrial ROS → ferroptosis induction; synergistic with GCS inhibition; spares noncancerous cells	[395]
Natural agents	Curcumin	Polyphenolic constituent of Curcuma longa (turmeric)	ACSL4-dependent PUFA-PL peroxidation; system Xc⁻ (SLC7A11) ↓; GPX4 ↓; iron metabolism (FTH1 ↓)	In vivo, in vitro	HepG2, SMMC−7721; Nude mice (HCC xenografts, i.p. 20 mg/kg)	↑ ACSL4 and PTGS2; ↑ Fe²⁺, MDA, lipid peroxides; ↓ GSH; ↓ SLC7A11, GPX4, GSS, FTH1; ferroptosis inhibitors (Fer−1, Lip−1, DFO) rescue cell death; ACSL4 knockdown blunts effects → curcumin drives ferroptosis via ACSL4 upregulation and suppression of the SLC7A11/GSH/GPX4 axis	[396]
Natural agents	Tannic acid (TA)	Plant polyphenol; mixture of gallotannins (incl. gallic, trigallic, m-digallic acids)	Iron metabolism / NTBI handling (Fe³⁺ chelation); autophagy–phagocytosis–mediated iron trafficking	In vitro	HepG2	Binds small ferric complexes without displacing ferritin/transferrin; forms self-assembled Fe³ ⁺–TA complexes taken up by phagocytosis; induces autophagy; mobilizes intracellular iron as Fe³ ⁺–TA for efflux; inhibits iron-stimulated HepG2 growth; Fe-TA detectable by MRI for influx/efflux imaging.	[397]
Synthetic small molecules	YL−939	Synthetic small molecule; PHB2 binder	PHB2 → ↑Ferritin (FTH1/FTL) → ↓Labile iron; inhibition of ferritinophagy (NCOA4/lysosome axis)	In vivo, in vitro	LO2 hepatocytes; C57BL/6J mice (APAP-induced acute liver injury)	Directly binds PHB2 to upregulate ferritin and block ferritinophagy, lowering intracellular iron and lipid peroxidation (↓MDA/ROS); broadly prevents ferroptosis triggered by erastin/RSL3/ML210 in cells; ameliorates APAP-induced acute liver damage (↓ALT/AST, ↓hepatic/serum MDA, ↑hepatic ferritin).	[398]
Natural agents	Genipin	Natural iridoid (aglycone of geniposide; from Gardenia jasminoides)	Ferroptosis inhibition via GPX4/xCT upregulation and ALOX15 suppression (arachidonic-acid metabolism)	In vivo, in vitro	Mice with CCl₄-induced acute liver injury; human hepatocytes L-O2 (erastin)	↑ GPX4 and xCT; ↓ ALOX15-launched lipid peroxidation and lipid ROS; protects from CCl₄ hepatotoxicity; Fer−1 mimics effect; GPX4 knockdown blunts protection; ALOX15 knockdown reduces cytotoxicity	[399]
Synthetic small molecules	Metformin	Small-molecule biguanide; approved anti-diabetic	Ferroptosis inhibition via xCT/GPX4/ACSL4 axis	In vivo, in vitro	AML12 hepatocytes (erastin, RSL3, PA/OA); db/db mice ± RSL3	↓ Ferroptosis: ↓ iron & lipid-ROS (C11-BODIPY, 4-HNE), ↑ GSH/GSSG; ↑ GPX4 & xCT, ↓ ACSL4; improves ALT/AST, TG/TC, histology; effects partially reversed by RSL3	[400]
Natural agents	α-Lipoic Acid (α-LA)	Endogenous/supplemental dithiol antioxidant; small molecule	Nrf2↑ (Keap1–Nrf2–ARE activation), NF-κB↓; broad oxidative stress & inflammation pathways (not a direct ferroptosis assay)	In vivo	Wistar rats; AuNPs (10 nm; 5 µg; 2.85 ×10 ¹¹ NPs) ± α-LA 200 mg/kg i.p., 7 days	Protects against AuNP-induced hepatotoxicity: ↓ ALT/AST/γ-GTT; ↓ MDA, TNF-α, IL−6; ↑ GSH, SOD, CAT; ↑ total & nuclear Nrf2; ↓ total & nuclear NF-κB p65; normalizes Bax/Bcl−2, cleaved caspase−3; improves histology	[401]
Natural agents	Arbutin (ARB)	Glucoside of hydroquinone, derived from Arctostaphylos uva-ursi (bearberry)	FTO/SLC7A11 axis; m6A methylation; GPX4; iron metabolism	In vivo, in vitro	HepG2 (OA/PA-induced steatosis); C57BL/6 mice (HFD-induced NAFLD); AAV-mediated hepatic SLC7A11 overexpression models	ARB binds & inhibits FTO → ↑ m6A methylation at 3′UTR of SLC7A11 → stabilizes SLC7A11 mRNA → ↑ SLC7A11/GPX4 expression, ↑ GSH, ↓ ROS, ↓ Fe²⁺ accumulation, ↓ MDA → suppresses ferroptosis and alleviates NAFLD progression (improved steatosis, glucose tolerance, insulin sensitivity)	[402]
Synthetic small molecules	CDDO (2-amino−5-chloro-N,3-dimethylbenzamide)	Small molecule, HSP90 inhibitor	HSP90 → CMA (via LAMP2A/Hsc70) → GPX4 degradation	In vitro	HT−1080, Erastin-induced ferroptosis	Erastin activates CMA, leading to GPX4 degradation and ferroptosis; HSP90 inhibition by CDDO or CMA blockade stabilizes GPX4 and suppresses ferroptosis; links necroptosis and ferroptosis regulation	[403]
Natural agents	(+)-Clausenamide ((+)-CLA)	Alkaloid isolated from Clausena lansium leaves	Keap1–Nrf2 pathway (Cys151 binding); GPX4/SLC7A11; lipid peroxidation (MDA, 4-HNE); Ptgs2	In vivo, in vitro	HepaRG, HepG2, SMMC−7721, Bel−7402; C57BL/6 mice (APAP overdose; erastin-induced DILI)	Directly binds Keap1 at Cys151 → blocks Nrf2 ubiquitination → ↑ Nrf2 stability & nuclear translocation → ↑ HO−1, NQO1, GCLM, GSH synthesis; restores GPX4 & SLC7A11; ↓ lipid peroxidation (MDA, 4-HNE, ROS); ↓ Ptgs2; prevents ferroptosis and protects against APAP/erastin-induced hepatotoxicity	[206]
Natural agents	Silibinin	Flavonolignan (major active of silymarin)	Iron handling & autophagy–mitophagy axis: ↓TFR1, ↑ferritin; blocks NCOA4-dependent ferritinophagy; restores PINK1/Parkin-mediated mitophagy → ↓labile Fe, ↓ROS/lipid peroxidation, ↑GSH	In vitro	HepG2; HL−7702 (immortalized hepatocytes)	Ethanol/acetaldehyde induce ferroptosis (↑Fe, ↑ROS, ↑MDA, ↓GSH). Silibinin reverses these, normalizes iron transport/storage, reduces LC3-II↑/p62↓ autophagy changes, rescues ferritin, and via PINK1/Parkin knockdown shows mitophagy is protective.	[404]
Natural agents	Tiliroside	Flavonoid glycoside (from oriental paperbush flower)	TBK1–p62(Ser349)–Keap1–Nrf2 axis: directly inhibits TBK1, ↓p62 S349 phosphorylation → ↓p62–Keap1 affinity → Keap1-mediated Nrf2 ubiquitination/degradation → ↓Nrf2 targets (GPX4, FTH1, G6PD) → ferroptosis; synergy with sorafenib (blocked by NAC, liproxstatin−1)	In vivo, in vitro	HepG2, Hep3B, SMMC−7721; Nude mice (subcutaneous & orthotopic HepG2 xenografts)	Induces lipid peroxidation/ferroptosis and significantly enhances sorafenib efficacy without obvious toxicity; direct binding to TBK1 confirmed (molecular docking & BLI).	[405]
Natural agents	Quercetin	Plant-derived flavonoid (3,3′,4′,5,7-pentahydroxyflavone)	Mitochondrial ROS–mediated ferroptosis	In vivo, in vitro	L−02 hepatocytes (FFA-induced steatosis); C57BL/6J mice (HFD-induced NAFLD)	Quercetin reduced mitochondrial ROS, suppressed lipid peroxidation, iron overload, and GPX4 downregulation → alleviated hepatic ferroptosis and lipotoxicity	[406]
Natural agents	Diosgenin	Aglycone of dioscin; plant-derived saponin	mTOR–FASN / mTOR–HIF−1α–RELA–VEGFA	In vivo, in vitro	HepG2 (FFA-induced steatosis); Sprague–Dawley rats (HFD-induced NAFLD)	↓ p-mTOR → ↓ FASN (lipogenesis) and ↓ HIF−1α/RELA/VEGFA (inflammation) → ↓ TG/TC & cytokines; mTOR overexpression abolishes benefits ⇒ mTOR-dependent	[407]
Natural agents	Nuciferine (Nuc)	Aromatic aporphine alkaloid from Nelumbo nucifera	PPARα signaling	In vivo, in vitro	AML−12 hepatocytes (RSL3-induced ferroptosis); HFD mice (NAFLD model); ApoE−/− mice (hypercholesterolemia/NAFLD model)	Activates PPARα → improves fatty-acid handling & suppresses ferroptosis (↓ lipid peroxidation/iron overload); PPARα inhibitor GW6471 blocks Nuc’s protection and increases iron, confirming pathway dependence	[408]
Endogenous protein/peptide	Caveolin−1 (CAV1) / CAV1 scaffolding domain peptide (CSD)	Endogenous membrane scaffolding protein; peptide (aa 82–101: DGIWKASFTTFTVTKYWFYR)	NOX4 → ROS → GPX4 axis; xCT/System xc⁻; ACSL4; mitochondrial ROS/iron	In vivo, in vitro	AML−12 hepatocytes (FFA-induced steatosis); C57BL/6J WT & CAV1-KO mice (HFD-induced NAFLD); Human NAFLD liver samples	CAV1 directly binds NOX4 → ↓NOX4 activity → ↓ROS/oxidative stress, ↑GSH, ↑GPX4 & xCT, ↓ACSL4, ↓LPO/MDA, ↓hepatic iron; restores MMP, reduces inflammation (IL−1β/IL−18/TNF-α) & fibrosis (α-SMA/COL1) → attenuates NAFLD injury	[409]
Nanomaterial	PAA@Mn₃O₄ nanoparticles (PMO)	Ultrasmall poly(acrylic acid)-coated Mn₃O₄ nanozyme; ∼6 nm, negative surface charge	ROS detoxification (CAT-, SOD-, GPX-like activity); ferritinophagy/iron mobilization; mTOR signaling	In vivo, in vitro	AML−12; L02 hepatocytes; C57BL/6J mice (APAP- and I/R-induced acute liver injury)	Internalized mainly via macropinocytosis → lysosomal enrichment → broad ROS scavenging (esp. OH•) → inhibit ferritinophagy (↓NCOA4 degradation, ↓ferritin turnover, ↓labile Fe²⁺) → preserve mTOR phosphorylation → suppress lipid peroxidation & ferroptosis → protect against APAP-induced and I/R-induced liver injury	[291]
Nanomaterial	Silica nanoparticles (SiNPs)	Engineered amorphous silica NPs	NCOA4-mediated ferritinophagy → labile Fe²⁺ ↑ → lipid peroxidation (PL-OOH); autophagy/lysosome axis	In vivo, in vitro	L−02 human hepatocytes; Rodent liver models	SiNPs trigger ferritinophagy (↑autophagosomes containing ferritin → lysosomal degradation) → ↑ intracellular/mitochondrial Fe²⁺, ↑ PL-OOH, ROS; mitochondrial membrane/cristae rupture; impaired lipid-peroxide repair; Fer−1 rescues cytotoxicity & LPO; BafA1 accumulates ferritin-autophagosomes; NCOA4 knockdown blocks ferritin degradation, lowers Fe²⁺/LPO, protects cells	[286], [410]
Nanomaterial	Silver nanoparticles (AgNPs)	PVP-coated AgNPs (∼5–8 nm); environmental-relevant dosing	Ferroptosis via iron overload & lipid peroxidation; MAPK/PPAR pathways; genes: Arrdc3, Txnip, Egfr; insulin/glucose metabolism link	In vivo	Adult zebrafish (90–120 dpf; AgNPs ± DFO exposure)	AgNPs ↑ Fe²⁺ & MDA, mitochondrial condensation/cristae loss; RT-qPCR ↑ Arrdc3/Txnip/Egfr; DFO rescues Fe²⁺/MDA and morphology → ferroptosis drives liver inflammation/dysfunction	[284]
Nanomaterial	Chitosan-coated selenium nanoparticles (CS-SeNPs)	Elemental Se⁰ NPs stabilized with chitosan; oral 0.25 mg/kg; in vitro 50 µM	GPX4–GSH axis; iron homeostasis (↓TfR1/HO−1, ↑FTH1); anti-oxidative stress	In vivo, in vitro	BRL−3A rat hepatocytes (RSL3-challenge); ICR mice (CdCl₂-induced liver injury, 3 mg/kg i.p.)	Restores GPX4 protein & GSH-Px activity; ↓Fe²⁺ and MDA; normalizes SLC7A11/GPX4/FTH1/FTL (↑) and ACSL4/HO−1/TfR1 (↓); reduces ROS; rescues mitochondrial membrane potential and cristae integrity; lowers ALT/AST & histologic injury	[290]
Nanomaterial	Astaxanthin (ASX) and ASX-loaded hollow mesoporous silica NPs (HMSN@ASX)	Carotenoid antioxidant; HMSN carrier improves solubility and hepatic delivery	Nrf2/HO−1 activation → ↓oxidative stress; ↑autophagy; ↓ferroptosis	In vivo, in vitro	L02 human hepatocytes (APAP challenge); Mice (APAP-induced liver injury)	Activates Nrf2/HO−1, enhances autophagy flux, suppresses lipid peroxidation/ferroptosis; HMSN@ASX boosts liver targeting and therapeutic efficacy vs free ASX	[204]
Nanomaterial	Manganese oxide nanoflowers (MnO₂Nfs)	Hierarchical MnO₂ nanoflowers; multienzyme mimic (CAT, SOD, GPX-like; SOD dominant)	ROS detoxification; ferritinophagy inhibition; p62–Nrf2–GPX4 axis activation	In vitro, in vivo	Hepatocyte models; Mice (APAP-induced acute liver injury)	Potent scavenging of H₂O₂, O₂•⁻, •OH; restores GSH; blocks ferritinophagy-driven iron release; activates p62–Nrf2–GPX4 antioxidative pathway; prevents APAP-induced ferroptosis and oxidative injury	[411]
Natural agents	Xanthohumol (Xh)	Prenylated chalcone from hops (Humulus lupulus)	Nrf2–xCT(SLC7A11)–GPX4 axis via Keap1 cysteine modification	In vivo, in vitro	HepaRG cells (erastin challenge); C57BL/6J mice (APAP-induced liver injury)	Covalently modifies Keap1 Cys151/273/288 → inhibits Nrf2 ubiquitination → Nrf2 stabilization & nuclear translocation → upregulates xCT & GPX4; lowers Fe²⁺, ROS, LPO (MDA/4-HNE), restores GSH/SOD, preserves ΔΨm; mitigates APAP/erastin-induced ferroptosis and liver injury	[412]
Natural agents	Rehmanniae Radix Praeparata (RRP) aqueous extract	Herbal decoction (main components: acteoside, rehmannioside D, 5-HMF)	Iron metabolism regulation: ↓ZIP14 & hepcidin (Hamp); ↑FPN (Slc40a1), ↑Cisd1; restores GPX4/GSH; reduces ROS & LPO	In vivo, in vitro	BRL hepatocytes (MCT- and erastin-induced ferroptosis); C57BL/6J mice (hepatic ischemia–reperfusion injury, 2.5–10 g/kg gavage)	RRP corrects iron overload by ↓iron uptake (ZIP14), ↑iron efflux (FPN), ↑mitochondrial iron utilization (Cisd1), ↓hepcidin; restores GSH, ↑GPX4, ↑Nrf2/Hspb1, lowers ROS, MDA, and apoptosis; siRNA-Hamp knockdown synergizes with RRP. Ferroptosis inhibitor	[413]
Synthetic small molecules	Ticlopidine	Thienopyridine antiplatelet (P2Y12 antagonist)	Broad anti-ferroptosis action: ↓iron overload, ↓lipid peroxidation; preserves mitochondrial function	In vivo, in vitro	HUH7 cells; C57BL/6J mice (hepatic ischemia–reperfusion injury, 40 mg/kg i.p. at −24h and −2 h)	In mice: ↓necrosis/fibrosis, ↓ALT/AST; ↓Prussian-blue iron, ↓MDA/4-HNE, ↓PTGS2; ↓Gr−1 infiltration. In cells: dose-dependent rescue from ferroptosis induced by GPX4 inhibitors (RSL3, JKE−1674, FIN56), xCT/GSH axis blockers (IKE, BSO, SSZ) and iron-raisers (artesunate/DHA); ↓Fe²⁺, partial ↓lipid-ROS (C11-BODIPY), ↑ΔΨm (TMRE).	[414]
Natural agents	Enteromorpha intestinalis polysaccharide (EIP)	Sulfated heteropolysaccharide (≈42.3 % sulfate; ≈37.3 % total sugars; rhamnose-rich; Mw ≈113 kDa)	SLC7A11/GPX4 antioxidant axis; iron metabolism (tf/tfr/fpn/fth); lipid peroxidation (MDA)	In vivo	Adult male zebrafish (ethanol-exposed ALD model; diet 0.1 % EIP for 30 days in 0.2 % EtOH water)	↓Hepatic Fe²⁺ and PB-positive iron granules; ↓MDA; ↑SOD/CAT/GPx/GSH/GSSG/GR; ↓GST; improves ALT/AST/GGT/ALP, TG/TC; Oil Red O: ↓hepatic lipid vacuoles; RT-qPCR: ↓tf/tfr/fpn/fth, ↑gpx4/slc7a11 → inhibition of ethanol-induced ferroptosis	[415]
Synthetic small molecules	Donafenib + GSK-J4	Donafenib: multi-RTK inhibitor (deuterated sorafenib derivative); GSK-J4: KDM6A/B histone demethylase inhibitor	↑ HMOX1 via NRF2/ARE pathway; ↑ Fe²⁺ accumulation; lipid peroxidation	In vivo, in vitro	Huh7, HCCLM3, PLC/PRF/5, HepG2, Hep3B, Hepa1–6; Patient-derived HCC organoids; Mouse models (xenograft, PDX, hydrodynamic orthotopic HCC)	Synergistic lethality: co-treatment hyperactivates HMOX1, increases labile Fe²⁺, ROS, and lipid peroxidation → ferroptosis. Blocked by HMOX1 inhibition (ZnPP, CRISPR KO). Enhancer–promoter looping at HMOX1 locus confirmed by CUT&Tag and 3 C.	[416]
Nanomaterial	Fe-MnO₂ nanosheets loading dihydroartemisinin (Fe-MnO₂/DHA)	Fe³ ⁺-doped MnO₂ nanosheets as carrier; DHA loaded (≈35 % drug loading); GSH-responsive, ROS-generating nanoplatform	GPX4 inhibition via GSH depletion; Fenton/Fenton-like ROS generation; DHA endoperoxide cleavage; ICD induction	In vivo, in vitro	Hepa1–6, HepG2; C57BL/6 mice (tumor-bearing HCC model)	GSH-triggered release of Fe²⁺/Mn²⁺ → ROS overproduction (•OH, free radicals); inactivation of GPX4; LPO and MDA accumulation; ferroptosis + apoptosis. Induces ICD (CRT exposure, HMGB−1 release, ATP secretion), ↑CD4⁺/CD8⁺ T-cell infiltration, macrophage M2→M1 repolarization, ↓Tregs → enhanced systemic antitumor immunity.	[417]
Synthetic small molecules	Rociletinib (ROC)	Small-molecule covalent inhibitor; repurposed EGFR TKI	ACSL4 (blocks PUFA-CoA synthesis → ↓lipid peroxidation/ferroptosis)	In vivo, in vitro	mouse models of ALI	Covalently binds ACSL4 at Cys170 → inhibits enzymatic activity → reduces AA-CoA formation, suppresses phospholipid peroxidation and ferroptosis → alleviates ALI	[418]
Natural agents	Berberine (BBR)	Isoquinoline alkaloid, major bioactive of Rhizoma Coptidis	Autophagy–lysosome pathway (ALP) and UPS → ferritin degradation → ROS/Fe²⁺ accumulation	In vivo, in vitro	HSC-LX2, HSC-T6, LO2 hepatocytes; Mice (TAA- and CCl₄-induced liver fibrosis)	BBR suppresses autophagy, enhances ROS production and ferrous release via ferritin degradation (UPS-mediated), leading to lipid peroxidation, GSH depletion, and ferroptosis of HSCs. Fer−1 reverses effects.	[419]
Natural agents	Palmitoleic acid (PA)	Monounsaturated fatty acid (macadamia nut oil–derived)	ACSL4–lipid peroxidation axis; GPX4/SLC7A11 antioxidant axis	In vivo	C57BL/6 mice, HFD-induced NAFLD (0.5 g/kg PA q48h; 16-week study)	PA alleviates NAFLD and inhibits ferroptosis: ↓ACSL4, ↓hepatic Fe²⁺/ROS/MDA, ↑GPX4/↑SLC7A11; improves dyslipidemia and insulin resistance; molecular docking supports PA–ACSL4 binding	[420]
Natural agents	Atractylodes lancea rhizome polysaccharide (AP)	Plant-derived polysaccharide (multi-MW fractions; arabinose/galactose/glucose/fructose-rich)	p53/mTOR–autophagy → ferroptosis; KEAP1/NRF2/NCOA4–FTH1/GPX4 antioxidant–iron axis	In vivo, in vitro	C57BL/6J mice, MCD diet NASH model; PPC as positive control; 3-MA autophagy inhibition	AP suppresses p53↑/mTOR↓-driven autophagy, thereby limiting autophagic ferroptosis: ↓Fe²⁺/ROS/MDA, ↑SOD/↑GSH; restores GPX4/FTH1, lowers KEAP1/NCOA4; reduces IL−1β/IL−6/TNF-α, steatosis and injury; 3-MA confirms autophagy precedes ferroptosis	[421]
Natural agents	Poria cocos polysaccharides (PCP)	Plant-derived polysaccharide mixture (glucose/fucose/arabinose/xylose/mannose/galactose)	NRF2/KEAP1→HO−1/GPX4/FTH1/GSH antioxidant–iron axis; NF-κB/MyD88 inflammatory axis; ferroptosis	In vivo, in vitro	SD rats, alcohol-induced ALD (gavage; 6 weeks); BRL3A hepatocytes; modulators: ML385 (NRF2 inhibitor), Fer−1	PCP activates NRF2 signaling (↑Nrf2/HO−1/GPX4/FTH1/GSH) → ↓Fe²⁺/ROS/MDA and steatosis; suppresses NF-κB (↓p65/p-p65/MyD88) → ↓IL−1β/IL−6; improves ALT/AST/GGT, TG/TC; effects blunted by ML385 and mimicked by Fer−1 → ferroptosis-dependent protection	[422]
Natural agents	Wogonin	Flavone monomer (from Scutellaria baicalensis)	ALOX15 / iNOS–mediated ferroptosis; lipid peroxidation axis	In vivo, in vitro	SD rat DCD liver transplantation I/R model; BRL−3A hepatocytes under OGD/R	Wogonin attenuates IRI and improves survival by suppressing lipid peroxidation and ferroptosis; bioinformatics+docking+CETSA identify ALOX15 and iNOS as direct targets; ALOX15/iNOS silencing phenocopies protection and negates wogonin’s effect → pathway specificity	[423]
Natural agents	Oleanolic acid (OA)	Pentacyclic triterpenoid; gut-microbial metabolite candidate	KEAP1/NRF2/ARE axis → anti-ferroptosis	In vivo, in vitro	SD rats (severe steatotic liver IRI); IAR20 (rat) & THLE−2 (human) hepatocytes (steatosis + hypoxia/reoxygenation)	OA upregulates NRF2 activity (↓NRF2 ubiquitination, ↑nuclear translocation; ↑NQO1/GCLC/GPX4), lowers Fe²⁺/MDA & lipid-ROS, restores GSH and mitochondria, thereby inhibiting ferroptosis and alleviating IRI; KEAP1 docking suggests competitive binding; effects blocked by NRF2 inhibitor (brusatol).	[424]
Natural agents	Schisandrin A	Bioactive lignan from Schisandra chinensis	AMPK/mTOR axis → ferroptosis defense (↑GPX4, ↑SLC7A11, ↑GSH)	In vitro	Huh7 hepatocellular carcinoma cells	Activates AMPK/mTOR signaling, upregulates antioxidant defenses (GPX4, SLC7A11, GSH), reduces lipid peroxidation and ferroptosis. Protective in oxidative/alkali injury models.	[425]
Regulators	GSTA1 and CTNNB1 inhibitors (Curzerene; β-catenin-IN−2)	Antioxidant enzyme (GSTA1) + transcription factor (CTNNB1/β-catenin); small-molecule inhibitors	GSTA1/CTNNB1 axis → peroxidase activity; GPX4/SLC7A11 regulation; ferroptosis suppression; sorafenib resistance	In vivo, in vitro	Huh7, HepG2, sorafenib-resistant HCC cells; patient-derived HCC organoids; BALB/c nude mice xenografts	GSTA1 peroxidase activity and CTNNB1 nuclear translocation suppress ferroptosis, promoting sorafenib resistance. Knockout or inhibition reinstates ferroptosis (↑MDA, ↓GSH, ↓GPX4/SLC7A11), sensitizes to sorafenib. Combination of GSTA1 inhibitor (Curzerene) + CTNNB1 inhibitor (β-catenin-IN−2) synergistically reduces tumor growth in cells, organoids, and xenografts.	[426]
Synthetic small molecule	SKF96365 (SOCE inhibitor)	Inhibitor of store-operated Ca²⁺ entry (SOCE) via STIM1	SOCE–CaN–NFAT axis → ↑SLC7A11 transcription → ↑GSH → ferroptosis resistance; sorafenib sensitivity	In vivo, in vitro	Hep3B, MHCC97H, HEK293T; sorafenib-resistant xenograft models (B-NDG mice)	STIM1 upregulation enhances SOCE, activates NFAT, upregulates SLC7A11, boosts GSH, suppresses ferroptosis → sorafenib resistance. SKF96365 blocks SOCE–CaN–NFAT signaling, downregulates SLC7A11, restores ROS/MDA accumulation, re-sensitizes HCC to sorafenib; effects reversed by Fer−1.	[427]

## 11 Targeting ferroptosis by mesenchymal stem cells (MSCs)

The potential of MSCs to alleviate liver damage has garnered significant attention due to the promising outcomes observed in both in vitro and in vivo studies [428], [429], [430]. Research indicates that MSCs and their exosomes can effectively prevent liver damage caused by ferroptosis, a type of cell death triggered by iron overload and lipid peroxidation [431], [432]. A study by Lin et al. (2022) demonstrated that treatment with MSCs and their exosomes prevented lipid peroxidation and the production of ROS, while regulating SLC7A11 (an anti-ferroptosis protein), leading to significant protective effects against liver injury and ferroptosis [17], [433].

In another study, MSC-exosomes incubated with baicalin significantly reduced ROS production and prevented ferroptosis [434], [435]. These effects were linked to the Keap1-NRF2 pathway and P62, which activate NRF2 and help protect against ferroptosis [436], [437]. Exosomes derived from heme oxygenase-1 (HO-1) modified MSCs showed the ability to reduce ferroptosis and protect liver tissue from IRI [433], [438]. These exosomes achieved this by delivering miRNA-204–5p, which targeted ACSL4, a key protein in ferroptosis [439], [440]. The study highlighted that miRNA present in MSC-derived exosomes could effectively target critical ferroptosis-related proteins, preventing ferroptosis-induced liver damage. For example, miRNA-214–3p targeted COX2 directly to prevent ferroptosis [434], [441], [442].Notably, MSCs protect against ferroptosis by increasing GPX4 and GSH levels and reducing ROS production. These mechanisms were confirmed through the use of ferrostatin-1 (a ferroptosis inhibitor), which supported the protective effects of MSCs [17], [353] (Table 2).

**Table 2:** Targeting ferroptosis by mesenchymal stem cells in rodents with liver injury.

Cell/Derivative	Source	Model	Dosing	Cargo / Target	Mechanism axis	Ferroptosis readouts	Functional outcomes	Ref.
MSCs ± MSC-Exo	Mouse compact bone–derived MSCs	CCl₄-induced ALI (C57BL/6J, male)	MSC: 5 × 10⁵ cells i.v. (6 h post-CCl₄); Exo: 8 mg/kg protein i.v.	CD44 / OTUB1 → SLC7A11 stabilization via deubiquitination	↑ System xC⁻ (SLC7A11/xCT), ↓ Ptgs2 (COX−2), ↓ LOXs, ↓ lipid-ROS	↓ C11-BODIPY lipid-ROS, ↓ MDA; WB/IF: ↑ SLC7A11	↓ ALT/AST; histopathological improvement; efficacy comparable to Fer−1	[17]
Baicalin-pretreated MSC-Exo (Ba-Exo)	Mouse BM-MSCs (baicalin preconditioned)	D-GalN/LPS-ALI (C57BL/6)	150 µg exosomes i.v. at 1 h post-insult	p62	p62–Keap1–NRF2 activation	↓Fe²⁺, ↓MDA, ↑GSH; WB/RT-qPCR: ↑GPX4 / SLC7A11, ↓5-LOX; ↓ROS (DHE)	↓ALT/AST, ↓TNF-α/IL−6/MCP−1, reduced hepatomegaly	[443]
HO−1-MSC-sEV (HM-sEVs)	Rat BM-MSCs (HO−1 modified)	Steatotic donor LT (SD rats)	2.5 × 10 ¹ ⁰ particles via portal vein, post-LT	miR−214–3p → COX−2	miR−214–3p–COX−2 axis	↑GPX4, ↓4-HNE; ↓MDA/Fe²⁺	↓IRI severity, ↓Suzuki score, ↓ALT/AST/TBIL; loss of effect with miR−214–3p knockdown	[441]
HO−1-MSC-Exo (HM-exo)	Rat BM-MSCs (HO−1 modified)	LT with steatotic donor + H/R hepatocytes	2.5 × 10 ¹ ⁰ particles portal vein	miR−124–3p → STEAP3	miR−124–3p–STEAP3 inhibition	↓LPO, ↓MDA/4-HNE, ↑GPX4	Reduced IRI; STEAP3 overexpression reversed protection	[442]
MSCs / MSC-Exo (hBMSC)	Human BM-MSCs	AOLT / hepatic IRI (rat) + human hepatocytes	MSCs i.v.; Exo co-culture	miR−16–5p → SLC39A14	SLC39A14 downregulation → ↓NTBI (Fe²⁺) uptake	↓Fe²⁺ (tissue), ↑NTBI (serum), ↓MDA/4-HNE, ↑GPX4	Reduced hepatocyte ferroptosis & IRI; effect reversed with target manipulation	[444]
HO−1/BMMSCs (cells)	Rat BM-MSCs (HO−1 modified)	Severe steatotic liver IRI (rat) + H/R hepatocytes	2 × 10⁶ cells (caudal/portal vein per protocol)	–	AMPK → NRF2 → FTH1	↑FTH1, ↑p-AMPK, ↑NRF2; blocked by Compound C or Nrf2-KD	Reduced IRI and ferroptosis; protection lost with AMPK/NRF2 inhibition	[445]
MSC-Exo (miR−26a mimic-Exo)	Human BM-MSCs (exosomes engineered with miR−26a)	CCl₄-fibrosis (mice, 8w) + LX2HSCs	In vivo: i.v. 100 µg/mL; in vitro: 50 µg/mL	miR−26a → SLC7A11 (in HSCs)	Pro-ferroptosis in HSCs (to suppress activation)	In HSCs: ↑ROS/Fe²⁺/MDA, ↓GSH; reversed by DFO	↓α-SMA/Collagen I, ↓fibrosis (Masson), ↓ALT/AST; overexp. SLC7A11 reversed effect	[446]

## 12 Other interventions targeting ferroptosis

Beyond classical categories such as natural products, synthetic agents, nanoparticles, and MSC-based therapies, several additional interventions have emerged that modulate ferroptosis and hold therapeutic promise for liver diseases. Fibroblast growth factor 21 (FGF21), a hepatokine involved in glucose and lipid metabolism, has recently been implicated in ferroptosis regulation. In models of iron overload–induced liver fibrosis, FGF21 activation enhances the Nrf2 pathway while simultaneously promoting ubiquitination and degradation of HO-1, thereby suppressing excessive heme metabolism and ROS formation. These actions restore redox balance, limit hepatocyte ferroptosis, and attenuate fibrotic progression, highlighting FGF21 as a potential endogenous anti-ferroptotic mediator [447], [448]. At the level of transcriptional and enzymatic regulation, glutathione S-transferase A1 (GSTA1) and β-catenin (CTNNB1) have been identified as key drivers of ferroptosis resistance, particularly in sorafenib-treated hepatocellular carcinoma. Both factors enhance peroxidase activity and sustain GPX4/SLC7A11 expression, blunting lipid peroxidation and enabling tumor survival under ferroptotic stress. Dual inhibition with curzerene (a GSTA1 inhibitor) and β-catenin-IN-2 (a CTNNB1 inhibitor) synergistically reinstates ferroptotic sensitivity, suppressing tumor growth in cell lines, patient-derived organoids, and xenograft models. This demonstrates that pathway-directed combinatorial inhibition may overcome ferroptosis resistance in HCC [426]. Caveolin-1 (CAV1), a membrane scaffolding protein, also contributes to ferroptosis modulation. Loss of CAV1 aggravates free fatty acid–induced oxidative injury and ferroptotic sensitivity in hepatocytes. Mechanistically, CAV1 directly interacts with NOX4, reducing ROS production, restoring mitochondrial membrane potential, and upregulating GPX4 and xCT expression while downregulating ACSL4. In high-fat diet–induced NAFLD mouse models and human NAFLD liver samples, CAV1 deficiency correlated with enhanced oxidative stress, inflammation, and fibrosis, whereas supplementation with a CAV1 scaffolding domain peptide restored ferroptosis resistance and improved hepatic outcomes [409]. Finally, ubiquitin-mediated regulation has emerged as a critical control point in ferroptosis. The HECT-type E3 ubiquitin ligase HUWE1 (also known as MULE) directly binds and ubiquitinates transferrin receptor 1 (TfR1), promoting its proteasomal degradation and thereby limiting iron uptake. Loss of HUWE1 stabilizes TfR1, expands the labile iron pool, and accelerates lipid peroxidation, while genetic or pharmacologic inhibition of TfR1 rescues this phenotype. Experimental evidence in hepatocyte-specific HUWE1-deficient mice and in human liver transplant biopsies supports its role as an endogenous suppressor of ferroptosis and a promising therapeutic target [377], [449]. In summary, these interventions, including hepatokines (FGF21), regulatory enzyme/transcription factor inhibitors (GSTA1 and CTNNB1), structural proteins (CAV1 and its scaffolding peptide), and ubiquitin ligases (HUWE1), illustrate the diversity of ferroptosis-modulating strategies beyond classical categories. They expand the therapeutic landscape by targeting upstream regulatory pathways, iron metabolism, and cellular redox balance, and may offer synergistic opportunities when combined with conventional ferroptosis inhibitors in the treatment of liver diseases.

## 13 Preclinical and clinical targeting ferroptosis in liver disease

Recent preclinical studies have demonstrated that inhibition of ferroptosis can attenuate liver injury, inflammation, and fibrosis, highlighting its potential as a therapeutic target [450]. In murine models of MASLD, ferroptosis inhibitors such as ferrostatin-1 and liproxstatin-1 have shown efficacy in reducing hepatic lipid accumulation, oxidative stress, and inflammatory responses. These agents not only prevent the progression of steatosis to steatohepatitis but also ameliorate liver fibrosis by restoring mitochondrial function and modulating macrophage polarization from pro-inflammatory M1 to anti-inflammatory M2 phenotypes [390]. Additional preclinical interventions targeting ferroptosis have expanded our therapeutic landscape. Compounds such as Nuciferine, diosgenin, NOX4 inhibitors, and vitamin D analogs have demonstrated hepatoprotective activity in murine or cellular models by upregulating PPARα, NRF2, or GPX4 pathways, thereby reducing oxidative stress, iron overload, and lipid accumulation [451], [452], [453], [454], [455]. Similarly, in ALD models, agents like Enteromorpha intestinalis polysaccharides and quercetin modulate ferroptosis-associated signaling to alleviate hepatic injury, while selenium supplementation synergizes with ferrostatin-1 to stabilize GPX4 and suppress ACSL4-mediated lipid peroxidation [415], [456], [457], [458]. Natural products such as wogonin, oleanolic acid, and Poria cocos polysaccharides also highlight the breadth of ferroptosis-targeted hepatoprotection across IRI and fibrotic models [422], [423], [424]. While these findings are promising, clinical translation remains in the early stages. Currently, several clinical trials are investigating agents that modulate ferroptosis-related pathways. For instance, antioxidants like vitamin E and its isoforms are in phase III/IV trials for NAFLD, aiming to counteract oxidative stress and lipid peroxidation. PPAR agonists, such as saroglitazar and lobeglitazone, are also under investigation, given their roles in lipid metabolism and potential to influence ferroptotic processes [163], [459]. Furthermore, Donafenib, a sorafenib derivative, has demonstrated superior survival benefits in HCC patients by enhancing ferroptotic stress through system Xc− inhibition, ROS amplification, and glutathione depletion [14], [228], [460], [461], [462]. Other natural and synthetic agents, including schisandrin, bavachin, curcumin, and iberverin, have shown ferroptosis-modulatory activity in hepatoma models, suggesting that ferroptosis modulation may serve as an adjunct strategy to overcome sorafenib resistance and reprogram tumor metabolism [396], [463], [464], [465], [466]. Increased preclinical research also indicates novel ferroptosis-driven therapeutics such as inhibitors of CTNNB1 and GSTA1, SOCE pathways inhibitors, and new nanocarriers such as GOA/miR nanocomplexes and MnO2 nanoflowers that enhance redox balance, recover GPX4 activity, and suppress iron deposition. These results unveil the translational possibility of ferroptosis modulation implementation into precision medicine strategies in liver diseases. Ultimately, balancing preclinical knowledge with paradigms of clinical trials will solidify ferroptosis as a valid therapeutic axis in MASLD, ALD, IRI, DILI, and HCC. Such synergy will require robust efforts in biomarker development, patient stratification regimes, and careful definitions of ferroptosis cross-talk with apoptosis, necroptosis, and other regulated death pathways. Despite of these developments, few direct clinical studies have specifically targeted ferroptosis in liver diseases. The complexity of ferroptosis as well as its interaction with other form of cell deaths requires additional studies to create specific, safe, and effective ferroptosis-focused therapy. Future clinical trials must confirm preclinical evidence and assess the therapeutic ability of ferroptosis modulation in liver disease patients.

## 14 Conclusion & limitation

Ferroptosis, a form of regulated cell death driven by iron overload and lipid peroxidation, has become recognized as a central mechanism in hepatotoxicity. Ferroptosis directly links iron metabolism, redox imbalance, and lipid remodeling, placing it at the intersection of liver pathogenesis and therapy. In this review, we summarized how ferroptosis contributes to a wide spectrum of liver diseases, including drug-induced, alcohol-associated, metabolic, ischemia–reperfusion injury, viral hepatitis, hepatocellular carcinoma, and other liver disorders. We emphasized its dual role: accelerating hepatocyte loss, fibrosis, and malignant progression on one hand, while offering a therapeutic opportunity for selectively targeting cancer cells and activated stellate cells on the other. We also outlined new therapeutic approaches, from natural compounds and synthetic molecules to stem cell–derived products and nanotechnology, that regulate ferroptotic signaling to restore antioxidant defenses, correct iron balance, or induce ferroptosis in tumor cells.

Despite promising progress, important challenges remain. Most current knowledge is based on animal and in vitro studies that only partially reflect human liver disease. Biomarkers used to identify ferroptosis, such as lipid peroxidation products or PTGS2 expression, lack specificity and overlap with other cell death pathways. The cellular diversity of the liver adds further complexity: protecting hepatocytes by inhibiting ferroptosis could at the same time limit beneficial ferroptotic removal of fibrogenic or malignant cells. In addition, the majority of ferroptosis-targeting agents remain in early experimental stages, with limited pharmacological and safety data. Finally, the tight connections between ferroptosis, oxidative stress, immune responses, and metabolic regulation make it difficult to isolate its exact role in disease progression.

Future work should focus on developing reliable biomarkers and imaging tools to track ferroptosis in patients, and on creating liver-targeted delivery systems to improve drug safety and precision. Carefully designed clinical trials are urgently needed to test ferroptosis modulators in human liver disease. Combining ferroptosis-targeted strategies with immunotherapy, anti-fibrotic agents, or metabolic interventions may prove especially effective. Ultimately, clarifying when ferroptosis acts as a harmful driver or a therapeutic opportunity will be key to translating these insights into safe and effective treatments for liver disease.

## CRediT authorship contribution statement

**Negar Hemmati:** Writing – review & editing, Writing – original draft, Visualization, Supervision, Software, Project administration, Methodology, Investigation, Data curation, Conceptualization. **Mahdieh Anoush:** Review & Editing, Conceptualization, Supervision. **Bahman Abedi Kiasari:** Review & Editing. **Alireza Torkamani:** Writing – original draft, Investigation, Data curation.

## Declaration of Competing Interest

The authors declare that they have no known competing financial interests or personal relationships that could have appeared to influence the work reported in this paper.

## Data Availability

Review article
